# Bottom-up computational design of shape-selective organic macrocycles for humid CO_2_ capture

**DOI:** 10.1038/s41557-025-01873-1

**Published:** 2025-07-22

**Authors:** Tao Liu, Hang Qu, Sam D. Harding, Isaiah Borne, Linjiang Chen, John W. Ward, Simon C. Weston, Andrew I. Cooper

**Affiliations:** 1 Materials Innovation Factory, Department of Chemistry, University of Liverpool, Liverpool, UK; 2 ExxonMobil Technology and Engineering Company, Annandale, USA; 3 Leverhulme Research Centre for Functional Materials Design, University of Liverpool, Liverpool, UK

**Keywords:** Computational chemistry, Crystal engineering

## Abstract

The capture of CO_2_ emissions using porous solids is challenging because polar water molecules bind more strongly in most materials than non-polar CO_2_ molecules. This is a challenge for both flue gas capture and for direct air capture alike. Here we develop a bottom-up computational screening workflow to calculate the binding energy of 27,446 diverse molecular fragments with both CO_2_ and water. Most molecules favour water binding, but bent, clip-like aromatic molecules exhibit potential for the desired reverse selectivity. This suggests that aromatic macrocycles with specific shapes can promote multiple weak *π*–*π* interactions with CO_2_ that surpass stronger but less numerous dipole–*π* interactions with water. We synthesize two water- and acid-stable molecular prisms with triangular and square geometries, as suggested by computation. Experiments confirm that the CO_2_ capture capacity of these prisms is unaffected by high relative humidity, surpassing the performance of benchmark commercial porous materials.

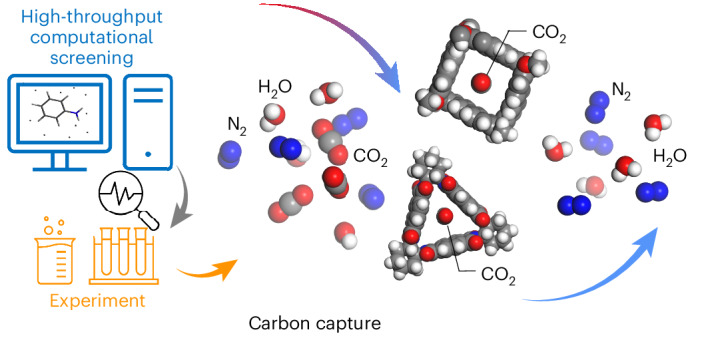

## Abstract (web summary)

Designing materials for CO_2_ capture under humid conditions is challenging due to the preferential binding of water in most porous adsorbents. Now it has been shown that shape-selective organic macrocycles, identified via bottom-up computational screening, can preferentially bind CO_2_ through multiple weak interactions, even in the presence of water.

## Main

Anthropogenic emissions of CO_2_ are a major contributor to global climate change^1,2^; however, capturing CO_2_ from dilute sources such as power plant flue gas presents considerable technical and financial challenges. Flue gas contains multiple components including nitrogen, water and oxygen. Some of the minority components can be removed by scrubbing before CO_2_ sorption, but water, which is typically present at high relative humidity, poses a greater challenge^3^. Drying flue gas before CO_2_ capture is prohibitively expensive^4^. Water is a strong competing adsorbate that reduces both the CO_2_ adsorption capacity and the regeneration efficiency of most adsorbents^5,6^. It is therefore imperative to develop adsorbent materials with high CO_2_ selectivity in wet flue gas.

Much effort has been devoted to developing solid adsorbents for efficient CO_2_ capture under both dry and humid conditions, including metal–organic frameworks (MOFs), zeolites and carbon materials^7–10^. Zeolites are important materials in the gas separation industry that have high CO_2_ uptakes; for example, zeolite Na-X (13X) and Ca-A exhibit dry CO_2_ uptakes of 3.38 and 3.81 mmol g^−1^ (313 K, 0.9 bar), respectively^11^. Porous carbon materials have been also investigated extensively for CO_2_ capture, including activated carbons, carbon nanotubes and graphene^12–14^; however, both zeolites and carbons are sensitive to moisture, and water leads to a substantial decrease in CO_2_ capacity and relatively poor selectivity^12,15,16^. Recently, MOF materials such as CALF-20 (ref. ^17^), IISERP-MOF2 (ref. ^18^) and MUF-17 (ref. ^19^) have demonstrated promising carbon capture abilities in wet flue gas, at least at moderate relative humidities (<40%)^20^. Aluminium formate (ALF) also functions at higher relative humidities^21^. The precise impact of water on CO_2_ sorption is not simple to predict. For example, CALF-20 (ref. ^17^) maintains consistent CO_2_ capture performance up to around 25% relative humidity. By contrast, zeolites 13X and Ca-A exhibit rapid deterioration in CO_2_ capacity under all humidities^11,15^, whereas *N,N*′-dimethylethylenediamine appended Mg-MOF-74 shows an increase in CO_2_ uptake at higher humidities owing to a chemisorptive cooperative insertion effect^22,23^.

High-throughput computational screening has emerged as a tool for targeting functional materials for gas adsorption and separation^7,24–26^. Computational screening can help to identify experimental targets and establish structure–property relationships, including the relationship between CO_2_ uptake and surface area or pore size. Although current datasets—such as those from the Cambridge Structural Database (CSD)^27^ and CoRE MOF (ref. ^28^)—include hundreds of thousands of porous materials, the diversity of organic building blocks that they represent is more limited, and skewed by synthetic and commercial availability. Furthermore, many MOFs incorporate polar organic linkers such as carboxylic acids and amines that might be expected to exhibit an inherent affinity for water. Indeed, coordinatively unsaturated sites in MOFs tend to prefer to bind water over CO_2_ due to the permanent dipole of H_2_O. Lin et al.^24^ conducted a comprehensive screening of about 600,000 zeolites and zeolitic imidazolate frameworks using the grand canonical Monte Carlo (GCMC) method, identifying promising materials for CO_2_ capture with low energy costs. Similarly, Boyd et al.^7^ simulated 300,000 MOFs, uncovering various classes of strong CO_2_ binding sites that offer good CO_2_ selectivity against N_2_ in humid conditions. Two water-stable MOFs (Al-PMOF and Al-PyrMOF), chosen for their hydrophobicity, were then synthesized, and it was found that humidity had a negligible influence on their CO_2_ capture performance up to 85% relative humidity; however, the performance of GCMC simulations of adsorption processes involving water is hard to control because of the poor accuracy of the force field parameters and the difficulty of modelling H_2_O adsorption in porous materials^29,30^. Hence, it is still challenging to perform large scale GCMC simulations of CO_2_/H_2_O selectivity in crystalline porous frameworks, and to capture the diversity of chemical space.

In this study, we stepped back from the problem of simulating CO_2_/H_2_O selectivity in porous frameworks and instead attempted to identify core molecular building blocks that might express this function in porous solids, given that local structures (building blocks, metals) are known to have marked effects on CO_2_ capture in the presence of H_2_O (refs. ^31,32^). Focusing on the molecular building blocks considerably reduces the computational cost. It is therefore possible to explore a wide range of chemical structures, perhaps identifying promising building blocks that have not been included in porous frameworks before. Furthermore, by focusing on building blocks rather than frameworks, we hoped to uncover learning that could be transferred to a range of porous materials beyond MOFs, such as porous polymers, covalent organic frameworks (COFs), porous molecular crystals and perhaps even porous liquids^33^.

We developed a computational workflow to screen a curated list of 27,446 unique molecular fragments by assessing their CO_2_ and H_2_O binding energies in isolation. This approach allowed us to identify fragments or motifs with superior CO_2_ binding affinity relative to H_2_O from a diverse range of chemical structures without the requirement for these fragments to be represented in porous materials. The chemical diversity of the organic building blocks screened surpasses that of the linkers represented in existing MOF/COF databases^27,28^. As expected, the vast majority of sampled molecules are predicted to bind H_2_O more strongly than CO_2_, re-emphasising the challenge of CO_2_ capture under humid conditions; however, some building blocks exhibit selective binding of CO_2_ against H_2_O. Bent clip-like aromatic fragments emerge, suggesting that exploting multiple weak *π*–*π* interactions between aromatic rings is a viable strategy. Subsequent calculations for hypothetical clips and prisms identify specific aromatic macrocycle ring sizes as being optimal for CO_2_/H_2_O selectivity owing to their size and shape. On the basis of these calculations, we synthesized two macrocyclic molecular prisms with triangular or square geometries. Dynamic column breakthrough (DCB) measurements confirmed that these structures maintained their CO_2_ capacities even in highly humid conditions, surpassing the performance of commercial benchmark materials.

## Results and discussion

### Computational screening (CHN dataset)

**Fig. 1:**
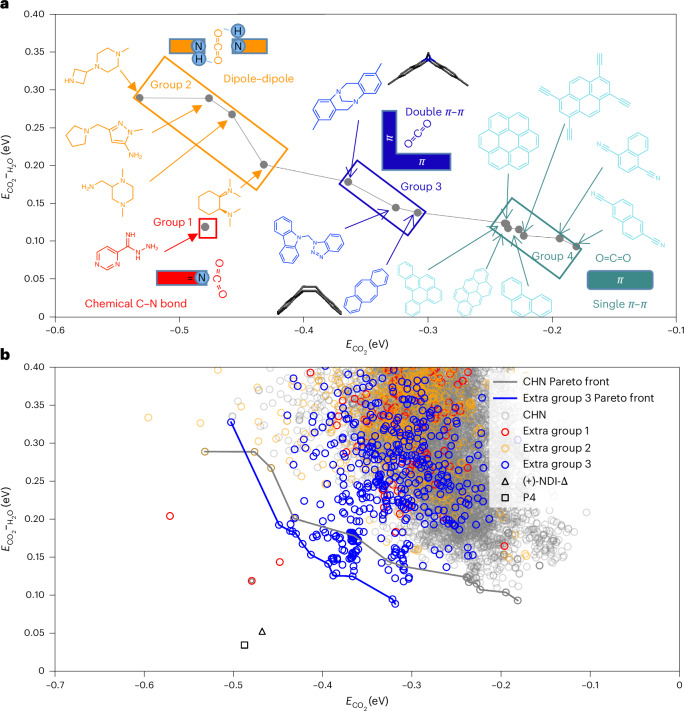
Computational screening for CO_2_/water selectivity using isolated molecular building blocks. **a**, Plot showing the Pareto front of calculated CO_2_ binding energy, $E_{\rm{CO}_2}$, versus CO_2_ against water, $E_{\rm{CO}_2-\rm{H}_2\rm{O}}$, for molecules in the CHN dataset (9,614 molecules in total, see Supplementary Fig. 2 for plot of full dataset; only data points with $E_{\rm{CO}_2-\rm{H}_2\rm{O}}$ < 0.40 eV are shown here). Note that chemisorptive amidines are essentially outliers, and not our focus here, so we have defined the Pareto front by non-amidine molecules. The highlighted molecular structures can be clustered into four groups: group 1, amidines; group 2, amines; group 3, aromatic molecular clips featuring tweezer-like aromatic regions at obtuse angles to one another. Two of these molecules are presented as 3D structures to illustrate the clip geometry. Grey, carbon; blue, nitrogen; hydrogen atoms are omitted for clarity; group 4, aromatic compounds. The shaded colourful boxes (red, orange, blue and cyan) represent how the molecules in groups 1, 2, 3 and 4 interact with CO_2_. **b**, 1,694 extra data points were added to the original CHN dataset by including additional molecules with high molecular similarity to those in groups 1–3. The grey line is the original Pareto front and is same as the Pareto front in Fig. 1a above. The blue line is the new Pareto front after the addition of this new dataset; the structures that advance the Pareto front are all clip-like (group 3). Triangles represent (+)-NDI-Δ, whereas square represent P4. Source data

The first molecular dataset—referred to here as CHN (9,614 entries)—comprises carbon, hydrogen and nitrogen elements exclusively, and was extracted from Reaxys (https://www.elsevier.com/solutions/reaxys; refer to Supplementary Fig. 1 for details on the high-throughput computational screening workflow used to calculate the binding energy between fragments and CO_2_ and H_2_O in gas phase). All 9,614 binding energy results from the CHN dataset are presented in Supplementary Fig. 2. Figure 1 shows the Pareto front, which emphasizes molecules with more favourable predicted CO_2_ binding energies ($E_{\rm{CO}_2}$) and CO_2_ selectivity against water, as estimated by $E_{\rm{CO}_2-\rm{H}_2\rm{O}}$, which is the difference between the maximum binding energy of the fragment with a single CO_2_ and H_2_O molecule, respectively. A smaller or more negative value for $E_{\rm{CO}_2-\rm{H}_2\rm{O}}$ indicates better selectivity. This screening methodology uses a single molecule of CO_2_ and H_2_O as the probe. It will not therefore capture any synergistic or antisynergistic effects between the multiple sorbate molecules; that is, it is more relevant to gas sorption at zero coverage.

Figure 1a shows that $E_{\rm{CO}_2}$ values range from −0.55 to −0.18 eV along the Pareto front, whereas all $E_{\rm{CO}_2-\rm{H}_2\rm{O}}$ values exceed 0.09 eV; that is, even the most favourable molecules in the CHN dataset are predicted to have greater binding energies with H_2_O than with CO_2_.

Further analysis reveals that the Pareto front can be categorized into four main groups based on distinct CO_2_ binding patterns, as follows:

Group 1: chemisorptive binding with an amidine (red structures).

Group 2: strong physisorptive binding with amines (orange structures), where multiple nitrogen atoms of the amine interact with the carbon atom of the CO_2_ molecule.

Group 3: physisorptive binding in a molecular clip-like structures (blue structures), where CO_2_ resides between and interacts with two aromatic arms at obtuse angles to one another, similar to a tweezer.

Group 4: physisorptive binding with aromatic compounds (cyan structures), where CO_2_ interacts with a single, flat aromatic fragment of varying sizes. Notably, pyrene units are rediscovered in this Pareto front, as found for extended MOFs by Boyd and co-workers^7^.

Figure 1a shows that as fragments exhibit stronger CO_2_ binding energies ($E_{\rm{CO}_2}$ values become more negative), they also tend to bind H_2_O more strongly ($E_{\rm{CO}_2-\rm{H}_2\rm{O}}$ values become more positive). This highlights the inherent difficulty of carbon capture from wet flue gas by physisorption. To drive the Pareto front towards the desired range (the bottom-left side), we expanded our dataset by adding 1,694 new fragments (a SMILES strings list is provided in the Supplementary Data 1) that have high molecular similarity to the fragments in groups 1–3 (Fig. 1b). Group 4 was excluded from this expansion owing to the impractically large number of similar aromatic molecules.

For group 1, most of the extra points (red circles) are inferior in predicted function to the parent data point. Only two additional data points in group 1 (bottom left-hand side of Fig. 1b) show possible good performance with respect to the parent data point (grey circle). One of these fragments exhibits desirable values for both $E_{\rm{CO}_2}$ (−0.48 eV) and $E_{\rm{CO}_2-\rm{H}_2\rm{O}}$ (0.12 eV) and is perfectly coincident with its parent (grey) datapoint due to a similar chemical structure to the parent amidine. Amidines are known to facilitate chemical CO_2_ capture^34^, and it is interesting that they also emerged from these unbiased computational screens in a bottom up way. For group 2, all of the extra data points (orange circles) are situated above and to the right of the original Pareto front (grey line), indicating no notable discoveries. The blue circles, representing extra structures obtained through similarity searches of the clip-like molecules in group 3, drive the original Pareto front (grey) further towards the desired region (blue line), leading to the discovery of more molecules with improved predicted performance—stronger CO_2_ binding and better CO_2_ selectivity against water than those found in the first fragment set (Fig. 1a).

In group 1, the CO_2_ molecule is absorbed chemically onto a nitrogen atom by forming a C–N bond between CO_2_ and the fragment, as reported previously for related amidines (for example, 1,8-diazabicycloundecene (DBU))^34^ or guanidines (for example, 1,3,4,6,7,8-hexahydro-2H-pyrimido[1,2-a]-pyrimidine)^35^. For amidines, CO_2_ capacities are likely to be limited, irrespective of the type of porous material that they are incorporated into, given that each amidine can chemically bind only one CO_2_ molecule. This strong covalent bonding also poses challenges for the regeneration process because of the large chemical binding energy, and we did not pursue amidines here.

In group 2, it is evident that CO_2_ can be captured through interactions with one, two or three nitrogen-attracting centres, while hydrogen atoms stabilize the oxygen atoms. However, as H_2_O is a more polar molecule than CO_2_ and forms stronger dipole–dipole interactions, enhancing CO_2_ selectivity against H_2_O remains difficult. Yet, unique patterns can be found to more strongly bind CO_2_ by arranging positive and negative charges^31^.

In group 4, we observed a slight increase (−0.1 eV) in $E_{\rm{CO}_2}$, by expanding the number of fused aromatic rings from 2 (naphthalene) to 4 (pyrene) or 6 (coronene), whereas the selectivity remains unchanged. Boyd et al.^7^ identified MOFs containing parallel aromatic rings as a promising candidate for CO_2_ capture in humid conditions through the screening of a large MOF structures dataset. We rediscovered pyrene-like structures and other fused aromatic molecules on the pareto front of our CHN dataset.

Our simulations involve only a single aromatic binding unit for the molecules in group 4. By contrast, the clip-like structures in group 3, with two aromatic rings working collaboratively to bind CO_2_, exhibit a different edge-to-edge arrangement compared to the experimental face-to-face arrangement reported by Boyd et al.^7^. We also observed that increasing the number of aromatic rings from one (group 4) to two (group 3) strengthens $E_{\rm{CO}_2}$ by approximately −0.1 eV because the additional aromatic ring expands the *π*-electron system and enhances dispersion as well as *π*–*π* interactions with CO_2_. Conversely, $E_{\rm{CO}_2-\rm{H}_2\rm{O}}$ remains nearly unchanged, differing by only 0.01 eV because water binding is driven primarily by hydrogen bonding at specific functional sites that remain largely unaffected by extra aromatic rings. This finding suggests a potential strategy for enhancing the selective capture of CO_2_ in porous materials under humid conditions, as explored further below.

To increase the compositional diversity of the datasets further, three additional datasets, named CHO (12,957 entries), CHF (344 entries) and CHNF (2,837 entries), underwent the same screening as the CHN dataset (Supplementary Fig. 3); however, we did not identify extra molecules with superior predicted performance because of hydrogen bonds between water and the molecules in these three libraries—some of which include alcohols, carboxylic acids and amides—that are much stronger than the interactions with CO_2_.

### Intermolecular interactions in group 3 (clip-like molecules)

**Fig. 2:**
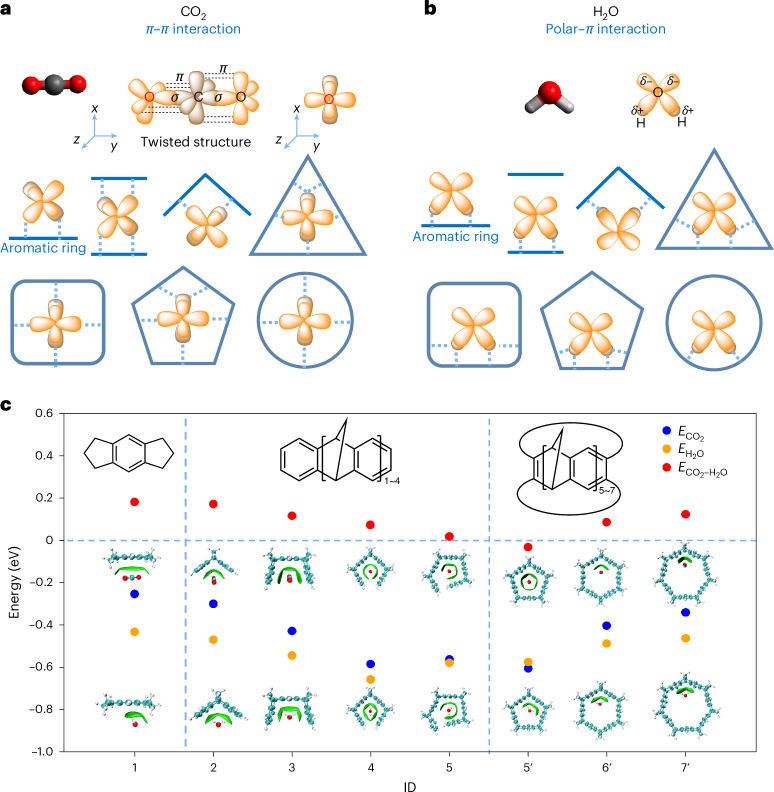
CO_2_ versus water binding selectivity. Selectivity arises from both the molecular symmetry of CO_2_ and water, and the size and shape of the molecular clips or macrocycles. **a**,**b**, The scheme shows interactions between CO_2_ (**a**) and H_2_O (**b**) with molecular clips and macrocycles of various sizes. **c**, Plot showing calculated $E_{\rm{CO}_2}$, $E_{\rm{H}_2\rm{O}}$ and $E_{\rm{CO}_2-\rm{H}_2\rm{O}}$, for known molecular clips ID 2/3/4/5 (ref. ^61^) and related hypothetical molecular models (ID 1/5′/6′/7′), together with their optimized structures with one CO_2_ or H_2_O molecule adsorbed. The green areas between the gas molecules and fragments represent their interaction regions, as analysed using the independent gradient model based on Hirshfeld partitioning; the isovalue is taken to be 0.002 e Å^−3^. Source data

The emergence of multiple promising candidate molecules in group 3 prompted us to study clip-like molecules in more detail. The oxygen atoms in the CO_2_ molecule form two C=O double bonds, each comprising one *σ* and one *π*-bond. Although the two *σ*-bonds are identical, the two *π*-bond hyperplanes are perpendicular to each other. This symmetry affects the binding interactions of CO_2_ with host molecules (Fig. 2a). When a CO_2_ molecule approaches a single aromatic ring, the *π* electrons of CO_2_ interact with the *π* electrons of the aromatic ring, forming *π*–*π* interactions, as depicted by dashed blue lines. When CO_2_ is surrounded by two aromatic rings (either face-to-face^7^, or in the molecular clips here), the two perpendicular *π*-electrons in CO_2_ interact with the *π*-electrons of two aromatic rings to form either double or quadruple *π*–*π* interactions. As the number of aromatic rings is increased from clips structures to macrocycles, it is possible to form a larger number of *π*–*π* interactions within the triangular, square, pentagonal or circular aromatic cavities.

By contrast, water interacts quite differently (Fig. 2b). H_2_O is a polar molecule with negative partial charges on the oxygen atom and positive partial charges on two hydrogen atoms. The resulting interaction between H_2_O and the aromatic ring(s) is characterized by polar–*π* interactions. As the number of aromatic rings increases, the hydrogen atoms of H_2_O still only interact with one or two aromatic rings at most. The number of interactions between H_2_O and the aromatic rings therefore does not increase with the number of rings in the same way that we observe for CO_2_. Again, this simple analysis does not account for any possible intermolecular interactions between sorbate molecules.

In general, *π*–*π* interactions are weaker than polar–*π* interactions due to the additional electrostatic component in the latter; however, multiple, cumulative weak *π*–*π* interactions might surpass stronger polar–*π* interactions by increasing their number. As such, net CO_2_ binding energies might exceed net H_2_O binding energies for certain molecular geometries containing multiple aromatic rings.

To validate the cooperative effect of multiple aromatic systems on CO_2_ affinity, we investigated a group of synthetically accessible molecules along with hypothetical model systems with varying numbers of aromatic rings (Fig. 2c). Across this range of molecules from ID = 1 to ID = 4, $E_{\rm{CO}_2}$ increases from −0.25 eV to −0.58 eV; $E_{\rm{H}_2\rm{O}}$ increases too, but less rapidly than $E_{\rm{CO}_2}$. Consequently, $E_{\rm{CO}_2-\rm{H}_2\rm{O}}$ improves continuously from 0.18 to 0.07 eV.

By increasing the number of surrounding aromatic rings in the molecular clip to 5 (ID = 5), $E_{\rm{CO}_2-\rm{H}_2\rm{O}}$ further improves from 0.07 eV to 0.02 eV; however, $E_{\rm{CO}_2}$ is not as negative as the clip with four aromatic rings because not all five of the aromatic rings in this twisted structure are fully interacting with the CO_2_ molecule. Furthermore, owing to molecular size and steric hindrance, it is challenging to design molecular clips with more surrounding rings. Hence, pentagonal, hexagonal and heptagonal molecular prisms (ID = 5′–7′) were investigated. The results indicate that the CO_2_ binding energy of the pentagonal structure (ID = 5′) is −0.61 eV. Moreover, $E_{\rm{CO}_2-\rm{H}_2\rm{O}}$ of this pentagon prism is −0.03 eV, indicating that a single CO_2_ molecule binds more strongly than a single H_2_O molecule in the prism cavity, unlike any of the 27,446 molecules from the initial screening study. In Fig. 2c the green areas between the gas molecules and fragments represent their interaction regions obtained by Multiwfn 3.8 (dev)^36–38^. There is clear evidence that CO_2_ interacts with five walls of the pentagonal prism, whereas H_2_O only interacts with three. Increasing the number of aromatic rings further to six (hexagon, ID = 6′) or seven (heptagon, ID = 7′) leads to a notable decrease in CO_2_ selectivity against water, despite maintaining an acceptable CO_2_ binding energy. This decline in selectivity and CO_2_ binding energy was attributed to CO_2_ not remaining in the middle of hexagon or heptagon prisms, resulting in a lower number of interactions with the aromatic rings, which agrees with the earlier theoretical results obtained by Ho and co-workers^39^.

### Computational screening (cyclic aromatic structures)

On the basis of these computational results, cyclic aromatic structures such as nanohoops, nanobelts and nanotubes are potential candidates for CO_2_ capture in humid conditions, as suggested in Fig. 2c, provided they have an appropriate size. Wang et al.^9^ reported that carbon nanotubes (CNTs) with cylindrical pore sizes between 7 and 8 Å would be suitable for post-combustion CO_2_ capture, based on the overlap of the potential energy surface of CO_2_ interacting with a cylindrical pore wall. They also noted that the binding strength of CO_2_ inside a CNT (with a diameter of 7.8 Å) is three times stronger than that on a single layer of graphene, corroborating our findings that the number of aromatic rings interacting with CO_2_ greatly influences CO_2_ adsorption; however, no measurement of CO_2_ capture performance in CNTs under humid conditions has been reported, nor did Wang et al.^9^ consider water as a competing species.

We screened a series of zigzag/armchair/chiral CNTs fragments with different diameters and found that zigzag (9,0) and armchair (5,5) exhibit the best selectivity for capturing CO_2_ under humid conditions (Supplementary Fig. 4). The CO_2_ binding selectivity against water diminishes as the size of the CNT increases; however, CO_2_ binding energies of CNTs (9,0) and (5,5), below −0.8 eV, are perhaps too strong to allow for the free diffusion of the flue gas, and could result in high regeneration costs. As such, (10,0), (11,0), (6,6) and (8,4) CNTs may represent a better trade-off, as they exhibit a more moderate CO_2_ binding energy (−0.6 eV) and a negative value for $E_{\rm{CO}_2-\rm{H}_2\rm{O}}$.

Nanobelts, which are short segments of CNTs, possess similar properties to CNTs, but may not present the same gas diffusion issues because of their greatly reduced length. We identified structures with diameters of 7–8 Å as promising candidates for CO_2_ capture under humid conditions including 5-cycloparaphenylene (5[CPP]), 6[CPP], [11]cyclacene, [10]cyclacene, [8]cyclacene, [10]cyclophenacene and Mobius molecules^40–43^—all of which are synthesizable in principle, albeit with often low synthetic yields.

### Synthesis of CO_2_-selective macrocycles

The computational results in Fig. 2c suggest that the increase in CO_2_ binding energy outpaces the increase in H_2_O binding energy as the number of surrounding aromatic rings varies, up to a total of around five aromatic rings (ID = 5′). We therefore sought synthesizable analogues of these molecules to test in the laboratory. Naphthalenediimide triangular prisms ((+)-NDI-Δ)^44^ and square anthracene pagoda[4]arenes (P4)^45^ were chosen as promising candidates. Although the pentagonal structure (ID = 5′) is the best candidate according to our simulation results, we are not aware of any report of its synthesis. There are reports of pillar[5]arenes with pentagonal shape^39,46^, but it is likely that the hydroxyl group in those molecules will bind water more strongly than CO_2_.

**Fig. 3:**
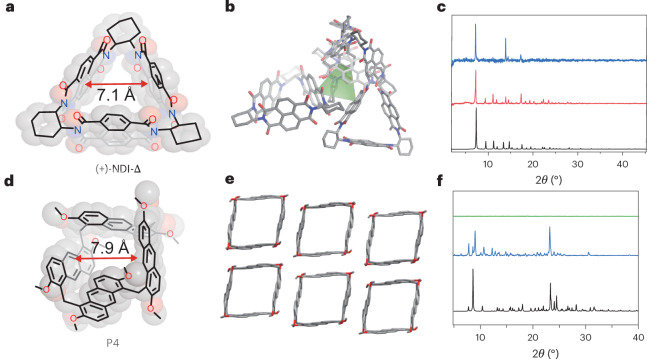
Experimental crystal structures of triangular and square macrocycles inspired by computational screening. **a**,**d**, Molecular and superimposed space-filling representations of (+)-NDI-Δ (**a**) and P4 (**d**). **b**,**e**, Crystal packing structures of (+)-NDI-Δ (**b**) and P4 (**e**) (see also Supplementary Figs. 8 and 9). Grey, carbon; blue, nitrogen; red, oxygen; hydrogen atoms are omitted for clarity. **c**,**f**, Powder X-ray diffraction patterns of (+)-NDI-Δ (**c**) and P4 (**f**). Black, simulated from single crystal structure; red, acetone-exchanged crystals; blue, activated materials; green, amorphous P4. Source data

Both (+)-NDI-Δ and P4 show uniform electron-rich prismatic cavities^47,48^ (Supplementary Fig. 5) with cavity diameters of 7.1 Å and 7.9 Å, respectively (Fig. 3a,d). The window diameter distributions of (+)-NDI-Δ and P4 were analyzed by pywindow^49^ based on the molecular trajectory obtained by xTB^50,51^ (Supplementary Fig. 5). The highest probability of the window size of (+)-NDI-Δ and P4 was 3.5 Å and 4.1 Å, which is close to the kinetic diameter of CO_2_ (3.3 Å). Furthermore, the robust nature of these two aromatic molecules holds promise for chemical stability under humid conditions. For example, the NMR spectra for these compounds were unchanged after immersion in water for two weeks (Supplementary Figs. 6 and 7).

In the single-crystal structures, (+)-NDI-Δ crystallizes in a tubular superstructure (Fig. 3b and Supplementary Fig. 8). No *π*–*π* stacking between the (+)-NDI-Δ molecules was found in the crystal; instead, four (+)-NDI-Δ molecules form an extrinsic tetrahedral packing void (green tetrahedron, Fig. 3b). Such voids may provide additional adsorption sites beyond the intrinsic prism cavities but reduce CO_2_/H_2_O selectivity, as our simulations show that at least two parallel aromatic side walls are required to enhance CO_2_ affinity. As such, the solid-state porosity for crystalline (+)-NDI-Δ is not solely defined by its triangular prismatic void. By contrast, the exo-wall *π*–*π* interactions between the anthracene units of adjacent P4 fabricates close-packed 2D rhombic tessellation in the solid state (Fig. 3a and Supplementary Fig. 9). The porosity in crystalline P4 is an almost perfect expression of the square macrocyclic cavity, as considered for a single P4 molecule in the gas phase.

The experimental powder X-Ray diffraction patterns of (+)-NDI-Δ and P4 are comparable with the simulated powder X-Ray diffraction patterns of their single-crystal structures (Fig. 3c,f). This indicates that (+)-NDI-Δ and P4 remain crystalline after exchanging the crystallization solvents; however, perhaps due to the instability of the extrinsic space among (+)-NDI-Δ molecules, this material remains only partially crystalline after activation (Fig. 3c; 358 K under vacuum for 12 h), whereas the close-packed P4 structure maintains high crystallinity after activation under the same conditions (Fig. 3f).

**Fig. 4:**
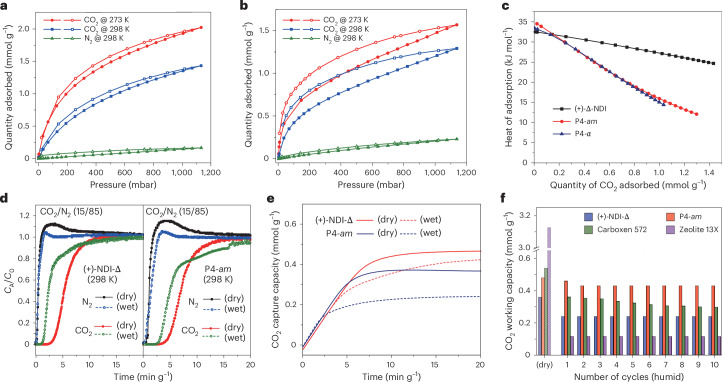
Gas sorption isotherms and dynamic column breakthrough separations for P4-*am* and (+)-NDI-Δ. **a**, CO_2_ (273 K and 298 K) and N_2_ (298 K) isotherms for (+)-NDI-Δ. **b**, CO_2_ (273 K and 298 K) and N_2_ (298 K) isotherms for P4-*am*. **c**, Calculated experimental isosteric heat of adsorption for CO_2_ on (+)-NDI-Δ, P4-*am* and P4-α. **d**, Experimental breakthrough curves for CO_2_/N_2_ separations under dry and humid conditions (75% relative humidity) for (+)-NDI-Δ (left) and P4-*am* (right) (85/15 v/v N_2_/CO_2_). *C*_0_ is the concentration of the corresponding gas in the inlet gas mixture, and *C*_A_ is its concentration at the outlet during breakthrough. **e**, Working CO_2_ capture capacity profiles for (+)-NDI-Δ and P4-am during breakthrough experiments under dry and humid conditions. The sorbent was pre-saturated by water; 75% relative humidity for humid conditions; 85/15 v/v N_2_/CO_2_; 298 K; 1 bar. **f**, Benchmarking the CO_2_ working capacity of (+)-NDI-Δ and P4-*am* against Carboxen 572 (a commercial activated carbon) and zeolite 13X under dry and humid conditions (pre-saturated by water for humid conditions, 85/15 v/v N_2_/CO_2_; 298 K; 1 bar) showing ten cycles of breakthrough experiments. Source data

The single-component gas sorption isotherms (N_2_, CO_2_) of activated (+)-NDI-Δ and P4 were collected and fitted using the Jensen–Seaton isotherm model^52^ (Supplementary Fig. 10). The CO_2_/N_2_ selectivity was calculated with ideal adsorbed solution theory (IAST) using pyGAPS^53^. The gravimetric CO_2_ uptakes of (+)-NDI-Δ was 1.93/1.35 mmol g^−1^ (273/298 K, 1 bar), whereas the N_2_ uptake was 0.14 mmol g^−1^ (298 K, 1 bar, Fig. 4a). The IAST selectivity of (+)-NDI-Δ (Supplementary Fig. 11) at 298 K is 25 for the adsorption equilibrium of the binary mixtures (CO_2_/N_2_:15/85 at 1 bar), which mimics the composition of the dry flue gas. Compared with (+)-NDI-Δ, crystalline P4 (P4*-α*, Supplementary Fig. 12a) has a somewhat lower CO_2_ capture capacity (1.21/0.98 mmol g^−1^, 273/298 K, 1 bar), but a steeper CO_2_ sorption curve is observed at low pressures (<0.2 bar). Rapid precipitation of P4 by addition of its dichloromethane solution into methanol affords amorphous P4 (P4*-am*; Fig. 3f), which exhibits a higher CO_2_ capture capacity (1.48/1.21 mmol g^−1^, 273/298 K, 1 bar) than crystalline P4-α. We attribute this to P4*-am* creating additional extrinsic pores in addition to the intrinsic cavities^54^. However, P4*-am* has similar CO_2_ uptakes to P4-α (Supplementary Fig. 12b) at lower pressure (<0.2 bar), suggesting perhaps that CO_2_ adsorbs preferentially in the intrinsic cavities of P4 at lower pressures. The zero-loading heat of adsorption (*Q*_st_) of both P4*-am* (34.9 kJ mol^−1^) and P4-α (33.4 kJ mol^−1^) for CO_2_ (Fig. 4c) is higher than that for (+)-NDI-Δ (32.6 kJ mol^−1^), reflecting the enhanced CO_2_ affinity of P4 due to its electron-rich square cavity at low coverages. Given its higher CO_2_ uptake and easier scalability compared with crystalline P4-*α*, we focused on P4-*am*, which also has a larger calculated IAST selectivity (~29) for CO_2_/N_2_ separations by comparing with (+)-NDI-Δ.

The maximum water uptake of (+)-NDI-Δ (Supplementary Fig. 13) is 3.45 mmol g^−1^ (298 K, *P*/*P*_sat_ = 0.97); this value is much lower than for zeolite 13X (18.2 mmol g^−1^) and, indeed, lower than for most reported microporous sorbents (Supplementary Fig. 14 and Supplementary Table 1). P4*-am* exhibited even lower water uptakes of 2.16 mmol g^−1^ under the same conditions, despite having higher porosity toward CO_2_. The single-component sorption isotherms for water and CO_2_ suggest that the CO_2_ selectivity against water predicted from isolated fragments (Fig. 2c) is expressed in these two macrocycles.

To further probe CO_2_ capture suitability of (+)-NDI-Δ and P4 for wet flue gas, we performed DCB experiments for a mixture of CO_2_/N_2_ (v:v = 15/85, 10 ml min^−1^, 1 bar, 298 K) under both dry and humid conditions (Supplementary Fig. 15). The simulated dry flue gas DCB experiments reveal both (+)-NDI-Δ and P4-*am* show good CO_2_/N_2_ selectivity (Fig. 4d). The CO_2_ working capacity for P4-*am* (0.48 mmol g^−1^) in dry conditions was consistent with the results of the equilibrium isotherm (0.49 mmol g^−1^ at 150 mbar), whereas (+)-NDI-Δ shows a slightly lower CO_2_ capture capacity (0.37 mmol g^−1^) than that in equilibrium isotherm (0.45 mmol g^−1^ at 150 mbar). For DCB experiments under humid conditions, all sorbents were pre-saturated by moisture before each run and between cycling experiments (Fig. 4f). This ensures that CO_2_ interacts with water-saturated material throughout the duration of the experiment, and the water is not removed by the gas flow. Our aim here was to simulate a worst-case scenario where water molecules have not been removed from the sorbent, recognizing that regeneration is possible, albeit with an energetic cost. The DCB traces for (+)-NDI-Δ exhibit a decrease in CO_2_ working capacity as the relative humidity increases from 0 to 75% (Fig. 4d,e, Supplementary Tables 2 and 3 and Supplementary Figs. 15–19), whereas humidity has little influence on the CO_2_ capture capacity of P4*-am*. These results validate the computational results, confirming that square prismatic macrocycles outperform triangular ones for CO_2_ capture selectivity under humid conditions.

Both (+)-NDI-Δ and P4-*am* show excellent recyclability and ease of regeneration. Cycling experiments at 75% relative humidity demonstrate moisture tolerance for both (+)-NDI-Δ and P4*-am*, with no substantial changes in working capacity after ten cycles and no observable degradation of the materials (Fig. 4f, Supplementary Fig. 20 and Supplementary Table 4). We further compared the performance of (+)-NDI-Δ and P4-*am* with two commercially available benchmark materials, zeolite 13X and an activated carbon (Carboxen 572). Zeolite 13X demonstrates a much higher capacity for CO_2_ capture (3.13 mmol g^−1^ at 15:85 CO_2_:N_2_) in dry flue gases, but this decreases markedly to just 0.12 mmol g^−1^ in the presence of moisture (Supplementary Figs. 16 and 17). Likewise, as the relative humidity increases, the CO_2_ capture capacity of Carboxen 572 decreases from 0.54 mmol g^−1^ to 0.39 mmol g^−1^, with a further decline observed after ten cyclic tests (Fig. 4f). It is worth noting that both (+)-NDI-Δ and P4 can accommodate only one CO_2_ molecule within their cavities and these cavities cannot be shared by multiple CO_2_ molecules simultaneously. Consequently, they exhibit low working capacities under dry conditions as compared to metal–organic framework materials such as Al-PMOF (ref. ^7^), Fe-MOR (0.25)^7,55^ and ALF (ref. ^21^); however, these materials still outperform well-known MOF materials at higher relative humidities (>75% relative humidity), including CALF-20 (ref. ^17^) and Al-PyrMOF (ref. ^7^). At these humidities, P4-*am* demonstrates uptakes comparable to recent high-performance CALF-20 variants, CALF-20M-e and CALF-20M-w (ref. ^56^).

We note that the direct comparison with gas breakthrough data (for example, Fig. 4c–f) reported in the literature is complicated by differences in methodology and, particularly, whether sorbents are pre-saturated with water before the breakthrough measurement^57,58^. Water velocity fronts travel much slower than lighter adsorbates, so pre-saturation with water is sometimes used to ensure that the entire adsorbent bed is humidified to draw conclusions about materials for CO_2_ capture in humid conditions^59^. The full regeneration of hydrophilic adsorbents requires parasitic energy. For carbon capture and storage, adsorbents must maintain or even increase their working capacities under humid conditions (for example, ALF (ref. ^21^), CALF-20 (ref. ^17^), IISERP-MOF2 (ref. ^18^), MUF-17 (ref. ^19^), Al-PMOF (ref. ^7^), *N,N*′-dimethylethylenediamine appended Mg-MOF-74 (refs. ^22,23^) and, here, P4). Hence, hydrophobic and water-stable macrocyclic materials such as P4 might be promising for industrial application, particularly when one considers potential regeneration strategies that involve passing steam over the sorbent, although the relatively modest CO_2_/N_2_ selectivity of P4 would need to be improved.

## Conclusion

Until now, the computational screening of structure–function properties in porous materials has focused on the porous frameworks themselves^7,24^. This has many advantages because it captures the effects of pore size and multiwall sorbent–sorbate interactions, but it has some limitations, too. First, periodic GCMC calculations are expensive, particularly for sorbate molecules such as water^29,30^. Second, the chemical diversity of computational screening using frameworks reflects the available framework chemistry, and it is dominated by organic linkers that produce MOFs (for example, aromatic carboxylic acids). Third, it is still challenging to create periodic simulations for amorphous porous solids.

Previously, Wilmer tackled this problem of computational cost by abstracting porous solids to an ensemble of Lennard–Jones spheres to create psuedomaterials^60^. This is a useful approach because it is computationally inexpensive and it can estimate the physical upper bound for adsorption properties in porous solids, such as high-pressure methane absorption. A limitation of pseudomaterials is that they do not capture specific directional sorbate–sorbent intermolecular interactions, nor it is simple to perform inverse design; that is, to translate a promising pseudomaterial into a real porous framework.

Our strategy here is a middle ground between psuedomaterials^55^ and full framework simulations^7,24^. The high-throughput workflow calculates the binding energies of CO_2_ and H_2_O, enabling the identification of optimal molecular fragments for carbon capture in humid conditions. These simulations are atomistic, but they are much less expensive than simulations for periodic frameworks. They are also not limited to molecular fragments that are known to produce porous frameworks. As such, this bottom-up fragment-based computational design strategy should also be applicable to materials beyond the shape-persistent ultrahydrophobic macrocycles discussed here, such as hydrogen-bonded organic frameworks, MOFs and COFs, as all of these materials are constructed from discrete organic building blocks.

This computational screen highlights the challenge of CO_2_ capture under humid conditions, as almost all of the 27,446 molecular fragments investigated were found to bind a single water molecule more strongly than a single CO_2_ molecule in the gas phase; however, we observed that multiple weak *π*–*π* interactions can lead to net CO_2_ binding energies that surpass the binding energy of more polar H_2_O molecules for specific numbers of aromatic rings. This led us to synthesize water-stable, hydrophobic triangular and square aromatic macrocycles, (+)-NDI-Δ and P4, that outperformed benchmark commercial materials for CO_2_ separation at high relative humidity. Future studies will focus on discovering related materials with larger pore volumes that retain this selectivity while exhibiting higher CO_2_ capacities. This bottom-up computational approach might also be transferable to designing materials for other industrially important separations, such as the separation of alkanes and alkenes.

## Methods

### Computational screening methodology

A computational sampling method was developed to directly calculate the binding energy between CO_2_, H_2_O and a given organic molecule in isolation. Initially, the self-consistent tight-binding method implemented in xTB^50^ was used for rapid structure screening. Density functional theory^62^ at the B97D3^51^ or Def2-SVP^63^ level was used for energy correction calculations; this was performed with Gaussian16^64^. Further details on the computational screening methodology can be found in Supplementary Fig. 1.

### Synthetic procedure for (+)-NDI-Δ

A solution of (*R*,*R*)-*trans*-1,2-cyclohexanediamine (0.88 g, 7.5 mmol) in DMF (10 ml) was added quickly to a solution of naphthalenetetracarboxylic dianhydride (1.49 g, 7.4 mmol) in DMF (100 ml) with vigorous stirring. The mixture was refluxed at 160 °C for 12 h. After the reaction completed, DMF was evaporated to dryness under reduced pressure (~3 mbar) at 75 °C. The crude product was purified by column chromatography over silica gel using CH_2_Cl_2_/acetone (0–10% volume). The crude product was recrystalized by using CH_2_Cl_2_ and MeOH to afford pure (+)-NDI-Δ (0.60 g) in 24% yield as a dark red solid.

### Synthesis procedure for P4

The mixture of 2,6-dimethoxyanthracene (1.8 g, 7.5 mmol) and paraformaldehyde (705 mg, 22.5 mmol) was dissolved in dichloromethane (600 ml). The solution was bubbled using N_2_ for 0.5 h, followed by the addition of TFA (304 μl, 4 mmol). The mixture was stirred at room temperature under N_2_ atmosphere for 12 h, water (400 ml) was then added to the flask to quench the reaction. The organic phase was separated and dried over anhydrous sodium sulphate and then evaporated to dryness. The residue was purified by column chromatography on silica gel (eluent: 1:1 petroleum ether/EA) to give P4 (521 mg, 27%) as yellow solids.

### Gas sorption methods

Before activation of the solvated crystals of (+)-NDI-Δ and P4, the crystallization solvents were exchanged with acetone and the resulting crystals were initially dried under a constant flow of N_2_ at room temperature. Crystalline (+)-NDI-Δ and P4 were degassed at 60 °C for 12 h under a dynamic vacuum before gas analysis. Single-component gas sorption isotherms (CO_2_ and N_2_) of (+)-NDI-Δ and P4 were collected using an ASAP2020 volumetric adsorption analyser (Micrometrics Instrument Corporation). Water isotherms of (+)-NDI-Δ and P4 were performed at 298 K using a Micromeritics 3flex surface characterization analyser, equipped with a Cold-Edge technologies liquid helium cryostat chiller unit for temperature control. CO_2_ isotherms for (+)-NDI-Δ and P4 were collected at 273 K, 288 K and 298 K to calculate the isosteric heat of adsorption for CO_2_. N_2_ isotherms for (+)-NDI-Δ and P4 were collected at 298 K.

### Dynamic column breakthrough experiments

Dynamic column breakthrough traces were collected using a Hiden Isochema ABR automated breakthrough analyser. The instrument was connected to a vapour generator set at 25 °C. A 2 ml stainless steel column (length = 10 cm; inner diameter = 0.5 cm) was packed with analyte (~0.4–1.3 g), glass wool was then added to both ends of the column to prevent any contamination to the system. The column was activated at the target temperature ((+)-NDI-Δ and P4, 60 °C; Carboxen 572 and zeolite 13X, 120 °C) for 12 h using helium as a purge gas before each experiment. Before recording CO_2_ breakthrough under humid conditions, the column was exposed to humid N_2_ at the desired relative humidity until saturated. Column breakthrough experiments were run under using CO_2_ in N_2_ (15/85, v/v) at a controlled temperature using a water bath at 25 °C with a steady post-column pressure of 1,000 mbar and a flow rate of 10 cm^3^ min^−1^. In the repeated cycling experiments, the samples were purged with helium flow for 2 h to remove the absorbed CO_2_ from material between each cycle. The samples were then presaturated with moisture for 3 h. A mass spectrometer (Hiden Analytical DSMS) was placed at the outlet to record the composition of the effluent gas. The equipment schematic is shown in Schematic **1**. Data was recorded at 3 s intervals using Isochema HIsorp 2017 (v4.01.0031) software.

Gas adsorption capacities were then calculated using the following equation:

1 $${\int }_{\!0}^{{t}_{\infty }}\left(1-\frac{F}{{F}_{0}}\right)d{t}_{\rm{sample}}-{\int }_{\!0}^{{t}_{\infty }}\left(1-\frac{F}{{F}_{0}}\right)d{t}_{\rm{blank}}=\frac{V\varepsilon }{{Q}_{0}}\left(1+\frac{1-\varepsilon }{\varepsilon }\frac{{q}_{\rm{ads}}}{{c}_{\rm{avg}}}\right)$$

Where *F* and *F*_0_ are the outlet and inlet flow rate of the adsorbate, respectively, *V* is the volume of the column, *ε* is the void space fraction, *Q*_0_ is the total volumetric flow rate at the inlet, *c*_avg_ is the average adsorbate concentration across the column and *q*_ads_ is the amount of adsorbate in the adsorbed phase at equilibrium.

## Online content

Any methods, additional references, Nature Portfolio reporting summaries, source data, extended data, supplementary information, acknowledgements, peer review information; details of author contributions and competing interests; and statements of data and code availability are available at 10.1038/s41557-025-01873-1.

## Supplementary information

Supplementary Information Supplementary Figs. 1–26, Tables 1–5, discussions, simulation and experimental details.

Supplementary Data 1 SMILES and binding energy results of CHN/CHO/CHF/CHNF datasets.

Supplementary Data 2 Optimized coordinates of NDI.

Supplementary Data 3 Optimized coordinates of P4.

Supplementary Data 4 Molecular dynamics initial structure of NDI.

Supplementary Data 5 Molecular dynamics final structure of NDI.

Supplementary Data 6 Molecular dynamics initial structure of P4.

Supplementary Data 7 Molecular dynamics final structure of P4.

## Source data

Source Data Fig. 1 Data to reproduce Fig. 1.

Source Data Fig. 2 Data to reproduce Fig. 2.

Source Data Fig. 3 Data to reproduce Fig. 3.

Source Data Fig. 4 Data to reproduce Fig. 4.

## Data Availability

Crystallographic data for the structures reported in this article have been deposited at the Cambridge Crystallographic Data Centre (CCDC), under deposition numbers CCDC 2384534 (NDI) and 2384535 (P4). Copies of the data can be obtained free of charge via https://www.ccdc.cam.ac.uk/structures/. All other relevant data generated and analysed during this study, which include experimental, spectroscopic, crystallographic and computational data, are available in the Supplementary Information and Supplementary Data. The list of SMILES representations of the structures used in high-throughput screening and their binding energy results are provided as Supplementary Data files. Source Data are provided with this paper.
