# Supplementary material for: Bottom-up computational design of shape-selective organic macrocycles for humid CO2 capture
Source: Nat Chem. 2025 Jul 22;17(11):1696–704. doi: 10.1038/s41557-025-01873-1 (PMC12580339; doi:10.1038/s41557-025-01873-1)
Supplement: Supplementary file 1 — Supplementary Figs. 1–26, Tables 1–5, discussions, simulation and experimental details. [file 41557_2025_1873_MOESM1_ESM.pdf]

nature chemistry

Supplementary information

https://doi.org/10.1038/s41557-025-01873-1

Bottom-up computational design of shape-selective organic macrocycles for humid CO_2 capture

In the format provided by the authors and unedited

1. Supplementary Methods

General information S3

Dataset and Computational methods S3

Gas sorption S3

Jensen-Seaton isotherm model S4

Single crystal X-ray diffraction (SC-XRD) S4

Dynamic column breakthrough (DCB) experiments S4

Adsorption kinetics S6

2. Supplementary Figures

Fig. 1 Workflow for grid search for screening CO_2 and H_2O binding energies for molecular fragments. S7

Fig. 2 Computational screening results for the CHN dataset (9,614 molecules) S9

Fig. 3. Individual Pareto fronts for the CHN, CHO, CHF and CHNF datasets S9

Fig. 4. E_{CO2}, E_{H2O} and $E_{CO2-H2O}$ for a series of zigzag, armchair, and chiral CNTs with varying diameters. S10

Fig. 5. Electrostatic potential map and calculated window size distributions for (+)-NDI-Δ and P4 S10

Fig. 6. Stability tests for macrocycles (+)-NDI-Δ under aqueous conditions S11

Fig. 7. Stability tests for macrocycles P4 under aqueous conditions. S11

Fig. 8. Single crystal structure of (+)-NDI-Δ S12

Fig. 9. Single crystal structure of P4. S12

Fig. 10. Curve fitting for gas sorption isotherms. S13

Fig. 11. IAST selectivity calculations. S13

Fig. 12. CO_2 sorption isotherms for crystalline and amorphous P4. S14

Fig. 13. Water sorption isotherms for the macrocycles. S14

Fig. 14. A comparison of materials with reported CO_2 (298 K, 1,000 mbar) and water (298 K, 32 mbar) isotherms in the literature. S15

Fig. 15. Experimental dynamic column breakthrough curves for macrocycles. S16

Fig. 16. Experimental dynamic column breakthrough curves for benchmark materials. S17

Fig. 17. Comparison of CO_2 breakthrough curves for macrocycles and benchmark materials. S17

Fig. 18. Kinetic fitting of adsorption profiles for (+)-NDI-Δ S18

Fig. 19. Kinetic fitting of adsorption profiles for P4-*am* S19

Fig. 20. Repeated cycling experiments S20

Fig. 21. ^{1}H NMR spectrum of (+)-NDI-Δ (400 MHz, $CDCl_3$). S21

Fig. 22. ^{13}C NMR spectrum of (+)-NDI-Δ (100 MHz, $CDCl_3$). S22

Fig. 23. ^{1}H NMR spectrum of P4 (400 MHz, $CDCl_3$). S23

Fig. 24. ^{13}C NMR spectrum of P4 (100 MHz, $CDCl_3$). S23

Fig. 25. Displacement ellipsoid plot for all non-H atoms in the single crystal structure of (+)-NDI-Δ (at 50% probability level) S25

Fig. 26. Displacement ellipsoid plot for all non-H atoms in the single crystal structure of P4 (at 50% probability level). S25

3. Supplementary Tables

Table S1. Table of CO_2 uptake against water uptake of materials with reported CO_2 (298 K, 1,000 mbar) and water (298 K, 32 mbar) isotherms in the literature S15

Table S2. Summary of CO_2 capture capacity S18

Table S3. Adsorption kinetics fitting parameters of CO_2 capture capacity of (+)-NDI-Δ and P4-*am*. S19

Table S4. CO_2 capture capacity of (+)-NDI-Δ, P4-*am*, Carboxen 572 and zeolite 13X in repeated cycling experiments (10 cycles). S20

Table S5. Crystal data and structure refinements S24

4. Synthetic procedures and NMR data for (+)-NDI-Δ and P4

Synthesis of (+)-NDI-Δ S21

Synthesis of P4 S22

5. Supplementary References

1. Supplementary Methods

General information: All chemicals were sourced commercially and used without further purification. NMR spectra (*i.e.*, ^{1}H- and ^{13}C-NMR spectra) were recorded at ambient temperature on a Bruker 400 NMR spectrometer (400 MHz and 100 MHz, respectively) and referenced against the residual ^{1}H or ^{13}C signal of the solvent. Powder X-ray diffraction (PXRD) data were collected in transmission mode using a Panalytical Empyrean diffractometer featuring a high throughput screening XYZ stage, an X-ray focusing mirror, and a PIXcel detector, utilizing Cu-Kα radiation (λ = 1.541 Å). The samples were placed on thin Mylar film in aluminium well plates for PXRD measurement.

Dataset and Computational methods:

CHN dataset (9,614 entries) comprises carbon, hydrogen, and nitrogen elements exclusively, and was extracted from Reaxys (REF). The molecules were chosen according to specific criteria, including molecular weight < 500 g mol^{-1}, number of elements (<= 3), charge (=0), number of rotational bonds (<=4), and availability from commercial suppliers, both to rationalize the size of the dataset and to bias it toward subsequent experimental accessibility. The complete SMILES strings list of the CHN dataset can be found in the Supplementary Data 1, along with the computed CO_2 and H_2O binding energies.

A sampling method was created to calculate the binding energy between CO_2, H_2O and a given molecule directly in five steps shown in supplementary Fig. 1. Initially, the Self-Consistent Tight-Binding (TB) method implemented in xTB[1] was used for rapid structure screening. Subsequently, Density Functional Theory (DFT)[2] on the B97D3[3]/Def2-SVP[4] level was used for energy correction calculations, performed by using Gaussian16;[5] see Supplementary Figure 1 and accompanying text for further details.

Gas sorption: Before activation of the solvated crystals of (+)-NDI-Δ and P4, the crystallization solvents were exchanged with acetone and the resulting crystals were initially dried under a constant flow of N_2 at room temperature. For all gas sorption experiments (+)-NDI-Δ and P4 were degassed at 60 °C for 12 hours under a dynamic vacuum prior to gas analysis. Single-component gas sorption isotherms (CO_2 and N_2) of (+)-NDI-Δ and P4 were collected using an ASAP2020 volumetric adsorption analyser (Micrometrics Instrument Corporation). Water isotherms of (+)-NDI-Δ and P4 were performed at 298 K using a Micromeritics 3flex surface characterization analyser, equipped with a Cold-Edge technologies liquid helium cryostat chiller unit for temperature control. CO_2 isotherms for (+)-NDI-Δ and P4 were collected at 273 K, 288 K and 298 K to calculate the isosteric heat of adsorption for CO_2. N_2 isotherms for (+)-NDI-Δ and P4 were collected at 298 K.

Jensen-Seaton isotherm model: The Jensen-Seaton isotherm model was used to describe the uptake of CO_2 and N_2 at 298 K, as follows:

$$q = KP\left[1+\left(\frac{KP}{a(1+\kappa P)}\right)^{c}\right]^{-1/c}$$

Where q (mmol g^{-1}) is the amount of adsorbate adsorbed, P (bar) corresponds to an equilibrium pressure, κ is adsorbed phase compressibility, K is the Henry constant, and c is an empirical constant. This equation has proven to be a better fit for experimental data from microporous solids compared to the Langmuir or Toth equations, especially for adsorbent/adsorbate systems with high Henry's constants. In these cases, the amount adsorbed increases rapidly at low pressures and then significantly slows down.

Single crystal X-ray diffraction (SC-XRD): Slow diffusion of methanol into a solution of (+)-NDI-△ (5 mg/mL) or P4 (5 mg/mL) afforded dark red hexagonal or yellow block crystals suitable for X-ray diffraction. SC-XRD data sets were collected on a Rigaku MicroMax-007 HF rotating anode diffractometer (MoKα radiation, λ = 0.71073 Å) or XtaLAB Synergy (Dualflex, HyPix, CuKα radiation, λ = 1.54184 Å). The raw data were reduced by CrysAlisPro 1.171.43.90. The structures were solved by the SHELXT[6] with Intrinsic Phasing and refined on F_2 by full-matrix least-squares methods with the SHELXL[6] and OLEX2[7] was used as GUI. The detailed crystal parameters are listed in the Supplementary Table S4 and the supporting CIFs.

Refinement details: all non-hydrogen atoms were refined anisotropically. Hydrogen atoms were placed at calculated positions using the riding model and refined isotropically. The instructions AFIX 23 and AFIX 43 were used for the hydrogen atoms on the secondary -CH2- and the aromatic C-H, respectively, with the parameter of Uiso = 1.2 Ueq. There are not any A- or B-level alerts in the crysatl structures of both (+)-NDI-△ and P4. For the crystal structure of (+)-NDI-△, a satisfactory disorder model for the solvent molecules was not found, therefore the PLATON/SQUEEZE routine was used to mask out the disordered density. A solvent mask was calculated and 3627 electrons were found in a volume of 8981 $Å^3$ in 3 voids per unit cell. This is consistent with the presence of 5[CH_2Cl_2], 1[CH_3OH] per formula unit which account for 3648 electrons per unit cell.

The absolute configuration of (+)-NDI-△ was assigned by reference to an unchanging chiral centre in the assembly procedure, i.e., the chiral carbon atoms of the reagent (S, S)-CHDA, instead of anomalous dispersion effects in diffraction measurements on the crystal and the derived Flack x parameter.

Dynamic column breakthrough (DCB) experiments: Dynamic column breakthrough (DCB) traces were collected using a Hiden Isochema ABR automated breakthrough analyser. The instrument was

connected to a vapour generator set at 25 °C. A 2 mL stainless steel column (length 10 cm; inner diameter 0.5 cm) was packed with analyte (0.4~1.3 g), glass wool was then added to both ends of the column to prevent any contamination to the system. The column was activated at the target temperature ((+)-NDI-△ and P4: 60 °C, Carboxen 572 and zeolite 13X: 120 °C) for 12 hours using He as a purge gas prior to each experiment. Before recording CO_2 breakthrough under humid conditions, the column was exposed to humid N_2 at the desired relative humidity until saturated. Column breakthrough experiments were run under using CO_2 in N_2 (15/85, v/v) at a controlled temperature using a water bath at 25 °C with a steady post-column pressure of 1000 mbar and a flow rate of 10 cm^3 min^{-1}. In the repeated cycling experiments, the samples were purged with He flow to remove the absorbed CO_2 from material between each cycle. The samples were then presaturated with moisture again, prior to re-analysis. A mass spectrometer (Hiden Analytical DSMS) was placed at the outlet to record the composition of the effluent gas. A schematic of the equipment is shown in Schematic **1**. Data was recorded at 3 second intervals using Isochema HIsorp 2017 software.

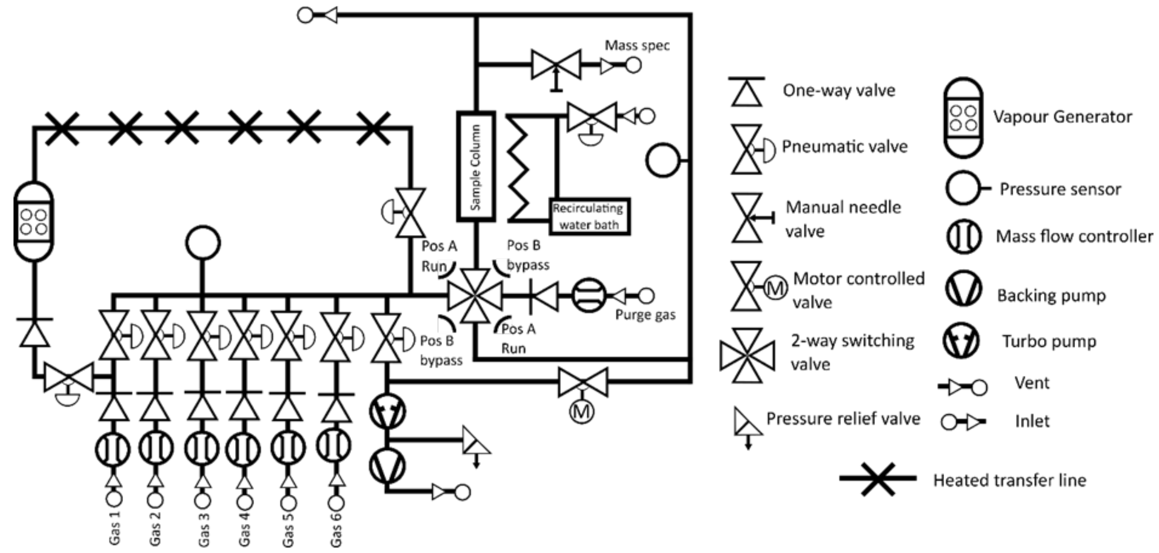

Schematic S1: Schematic diagram of the breakthrough apparatus.

Gas adsorption capacities were then calculated using the following equation:

$$\int_0^{t_\infty}\left(1-\frac{F}{F_0}\right)dt_{sample}-\int_0^{t_\infty}\left(1-\frac{F}{F_0}\right)dt_{blank}=\frac{V\varepsilon}{Q_0}\left(1+\frac{1-\varepsilon}{\varepsilon}\frac{q_{ads}}{c_{avg}}\right)$$

Where F and F_0 are the outlet and inlet flow rate of the adsorbate, respectively, V is the volume of the column, ε is the void space fraction, Q_0 is the total volumetric flow rate at the inlet, c_{avg} is the average adsorbate concentration across the column, and q_{ads} is the amount of adsorbate in the adsorbed phase at equilibrium. Adapted from Wilkins *et al.*[8]

Adsorption kinetics: The adsorption kinetics of CO_2 working capacity for both (+)-NDI-△ and P4 were analysed using Lagergrens pseudo-first-order (PFO) model and the pseudo-second-order (PSO) model. The Lagergren PFO model can be expressed as follows:

$$q(t)=q_e(1-e^{-k_1 t})$$

where q(t) and q_e are the amount of adsorbate adsorbed at any given time (t) and at equilibrium, respectively. k_1 denoted as adsorption kinetic rate constant in this model. The PFO model is particularly suitable for describing reversible interactions between the adsorbent and adsorbate, *i.e.*, the CO_2 adsorption behaviour on physical adsorbents.

The PSO model, on the other hand, assumes that the adsorption rate is proportional to the square of the number of available adsorption sites on the adsorbent surface. Its mathematical expression is given by:

$$q(t)=\frac{q_e^{\,2}k_2 t}{1+q_e k_2 t}$$

where k_2 is the rate constant of PSO adsorption. PSO model is often used to describe the adsorption behaviours that involve the formation of chemical bonds between adsorbate and adsorbent surface.

2. Supplementary Figures

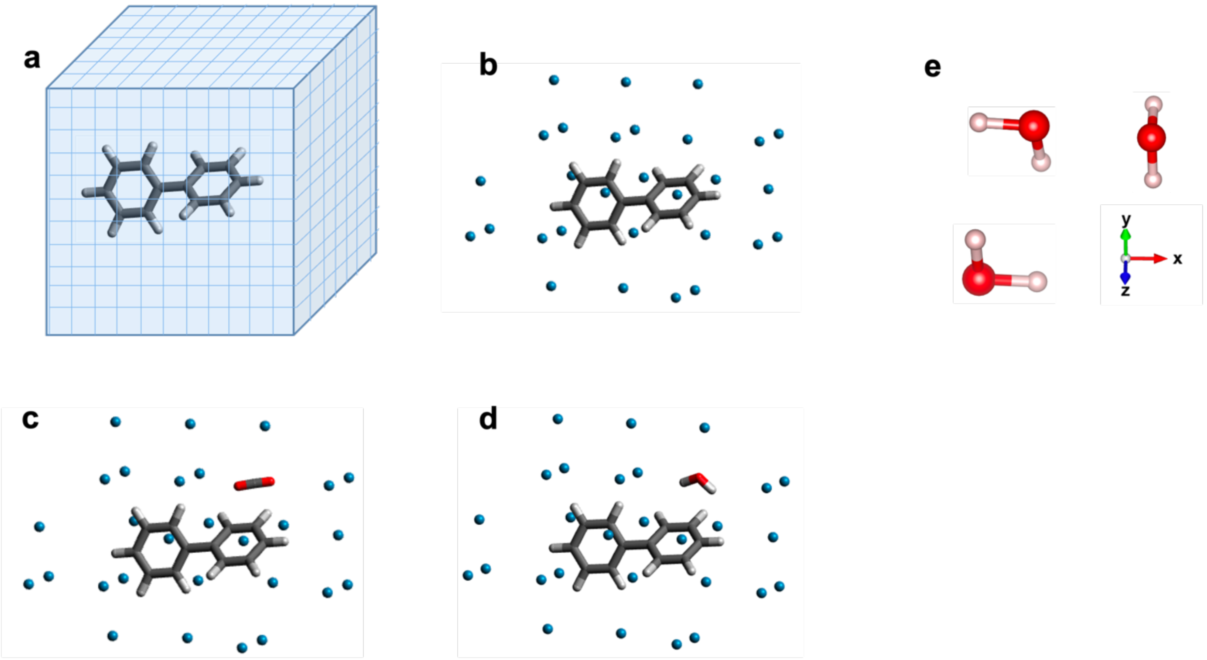

Supplementary Fig. 1. **Workflow for grid search for screening CO_2 and H_2O binding energies for molecular fragments. a**, Build a grid box for a given molecule, in this example case, biphenyl (grid resolution = 7x7x7). **b**, Retain the grid points that are between 2.5 and 5.5 Å from the nearest atom of the molecule and remove any points out of this range. **c**, Populate CO_2 on every retained grid sampling point and find the most stable CO_2 binding site. **d**, Replace the CO_2 molecule at the most stable CO_2 binding site obtained in **c** with a H_2O molecule with three different orientations, **e**. The overall workflow is as follows:

***Step 1*. Generation of 3-D molecular structures.** Starting from SMILES[9–11] strings for the fragment, the 3-D molecular structures and coordinates are generated and optimized by using UFF forcefield[12] implemented in RDkit[13] followed by geometry optimization by tight-binding method to get the energy of this molecule, $E_{molecule}$.

***Step 2*. Sampling points.** Construct a cubic grid box to enclose the given molecule, as shown in Supplementary Fig. 1a. The size of the molecule is defined by the distance between its two furthest atoms (maxDistance). The side length of the cubic grid box (lattice) is determined as maxDistance + a buffer of 8.0 Å. Position the centroid of the molecule at the centre of the grid box. Grid points are spaced at 4 Å intervals. Next, retain only the relevant points by calculating the distance between each grid point and the nearest atom of the molecule, as shown in Supplementary Fig. 1b. The relevant points are those with a distance between 2.5 and 5.5 Å from the nearest atom of the molecule.

***Step 3*. Strongest CO_2 binding site.** Embed one CO_2 molecule on every sampling point obtained in *Step2*, only one CO_2 orientation is considered because of the symmetry of CO_2 molecule. Relax the structure using tight-binding methods to find the strongest CO_2 binding site with lowest energy:

$E_{molecule+CO2}$.

$E_{CO2} = E_{molecule+CO2} - E_{molecule} - E_{CO2\_gas}$

where E_{CO2_gas} is the energy of a single CO_2 molecule in the gas phase.

E_{CO2}, the binding energy between target molecule and CO_2, is an indicator of CO_2 adsorption performance; the more negative, the stronger.

A list of molecules with $E_{CO2} < -0.2$ eV is passed to *Step 4*. (That is, we only determine water binding for fragments with acceptable CO_2 binding energies).

***Step 4*. H_2O binding energy.** Replace CO_2 with a H_2O molecule with three different orientations (Supplementary Fig. 1e) and optimize using tight-binding methods to obtain the energy of the most stable orientation, $E_{molecule+H2O}$.

$E_{H2O} = E_{molecule+H2O} - E_{molecule} - E_{H2O\_gas}$,

E_{H2O_gas} is the energy of a single H_2O molecule in the gas phase.

E_{H2O}, the binding energy between the molecule and H_2O, is an indicator of H_2O adsorption performance, the more negative, the stronger the interaction.

$E_{CO2\text{-}H2O} = E_{CO2} - E_{H2O}$

$E_{CO2\text{-}H2O}$ is an indicator of selectivity, or preference of the isolated fragment for CO_2 over H_2O; the smaller/more negative, the better.

***Step 5*. Accurate binding energy.** The structures obtained from *Step1/3/4* with $E_{CO2} < 0$ eV and $E_{CO2\text{-}H2O} < 0.1$ eV were reoptimized using DFT methods with B97D3 functional and def2-svp basis set to obtain accurate binding energies E_{CO2}, E_{H2O}, and $E_{CO2\text{-}H2O}$, which are the values reported throughout the paper, unless otherwise stated.

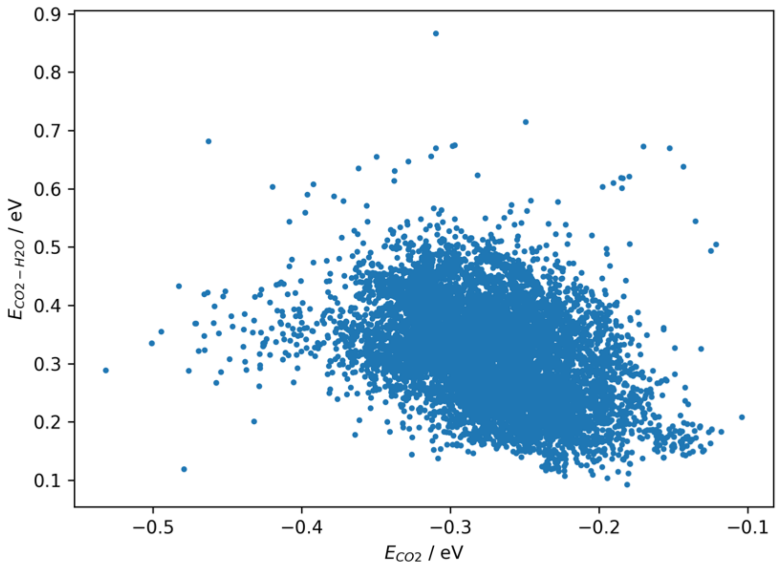

Supplementary Fig. 2. Computational screening results for the CHN dataset (9,614 molecules). $E_{CO2-H2O}$ is plotted as a function of E_{CO2} for the entire CHN dataset; only molecular fragments with E_{CO2} < 0.4 eV are shown in Figure 1 in the main text.

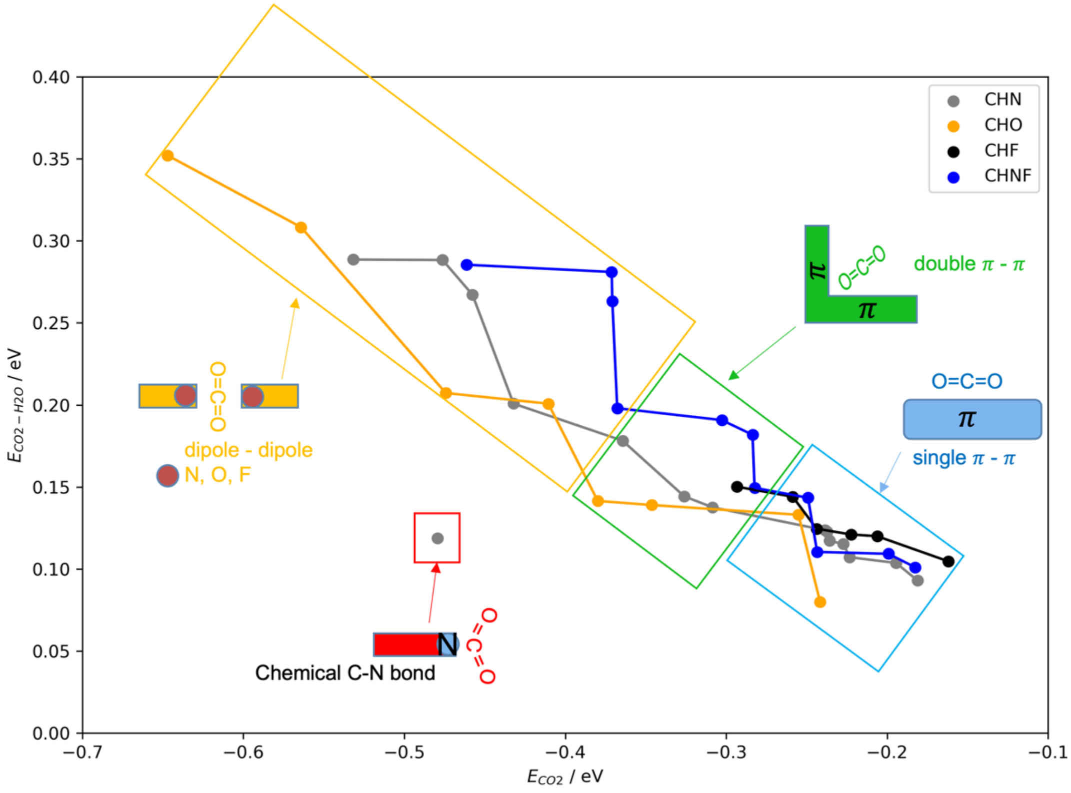

Supplementary Fig. 3. Individual Pareto fronts for the CHN, CHO, CHF and CHNF datasets. Red box: chemisorption (amidine); Yellow box: dipole-dipole physisorption (amines); Green box: double π-π physisorption (molecular clips); Blue box; single π-π physisorption (aromatic compounds). Molecular clips and macrocycles analogues were the key focus here; there is an inherent trade-off between binding energy selectivity, $E_{CO2-H2O}$, and CO_2 binding energy, E_{CO2}, as illustrated by the slope of the Pareto fronts. Chemisorptive amidines are effectively outliers, and excluded from the Pareto fronts for physisorptive molecular fragements.

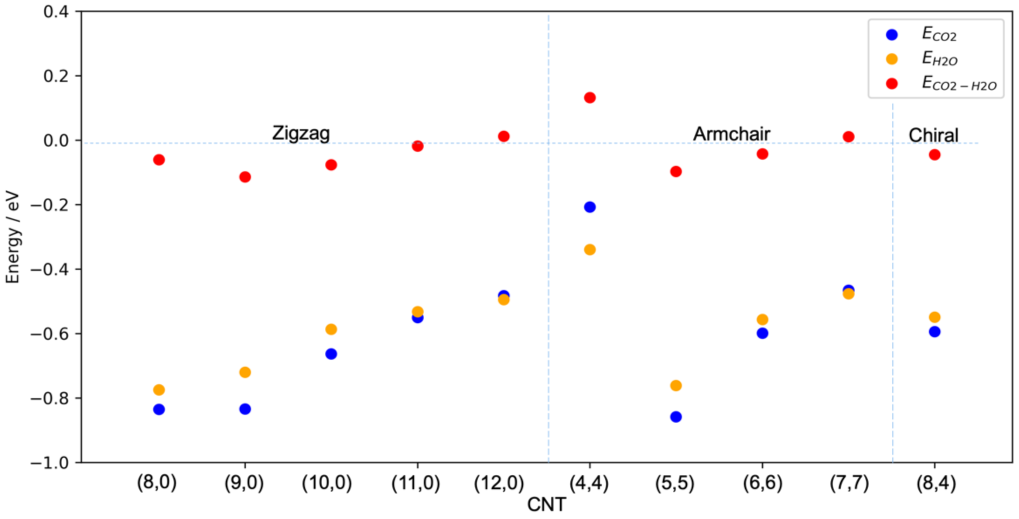

Supplementary Fig. 4. **E_{CO2}, E_{H2O} and $E_{CO2\text{-}H2O}$ for a series of zigzag, armchair, and chiral CNTs with varying diameters.**

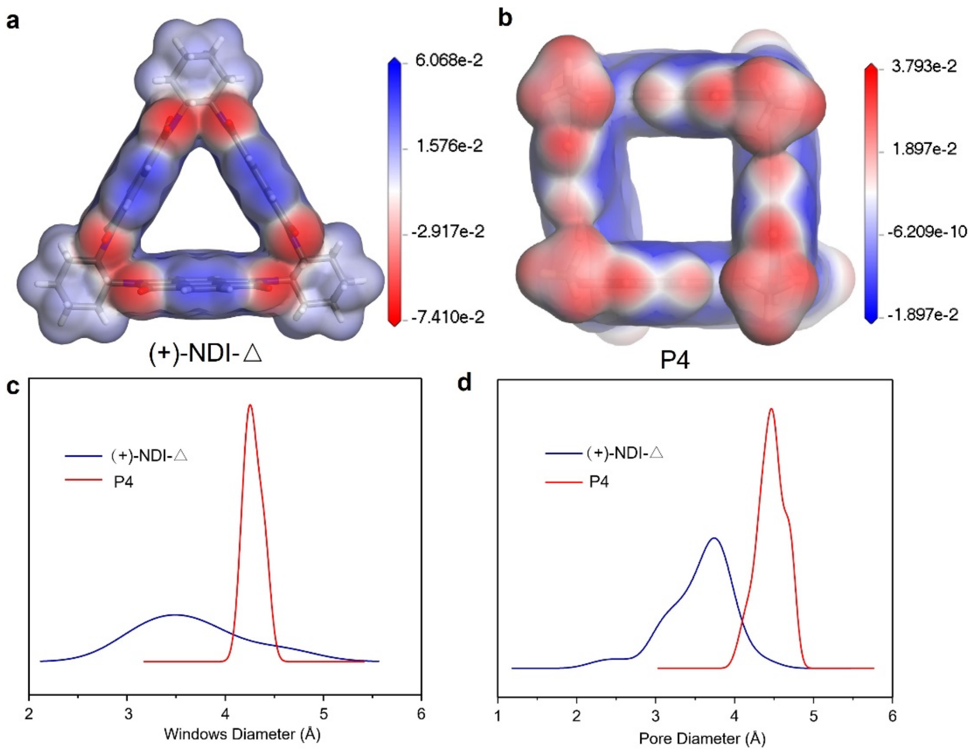

Supplementary Fig. 5. **Electrostatic potential map and calculated window size distributions for (+)-NDI-Δ and P4. a,** (+)-NDI-Δ and **b,** P4 show uniform electron-rich prismatic cavities; the aryl rings face the cavity interior. **c**, Window and **d**, pore diameter distribution histograms of (+)-NDI-Δ and P4 simulated by pywindow.

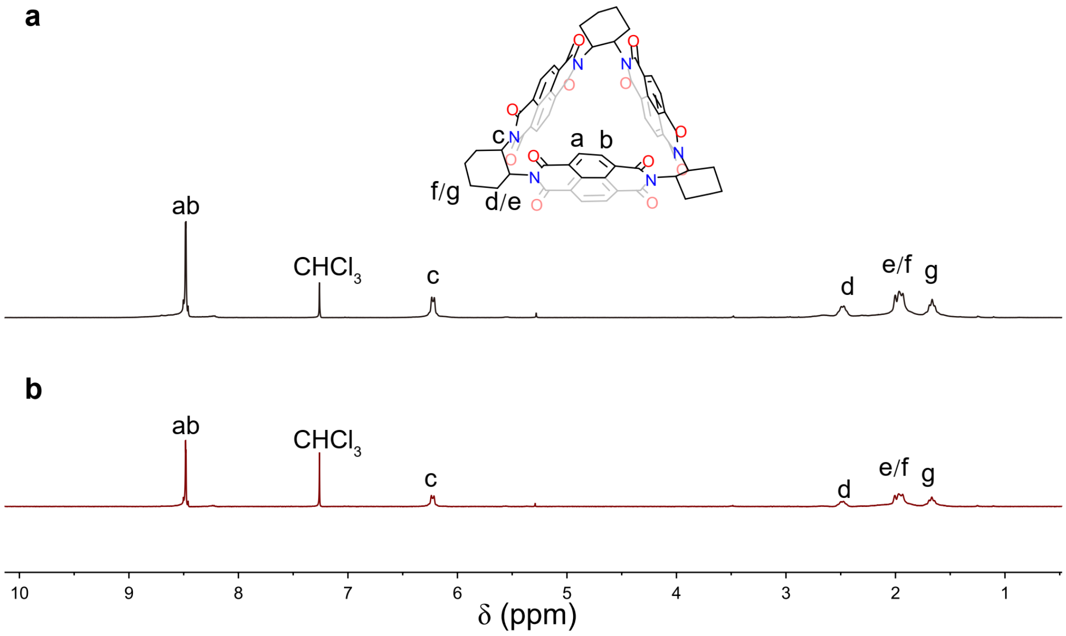

Supplementary Fig. 6. Stability tests for macrocycles under aqueous conditions. ^{1}H NMR spectra of (+)-NDI-Δ after immersion in water for two weeks (**a**) and **b**, (+)-NDI-Δ (400 MHz, $CDCl_3$).

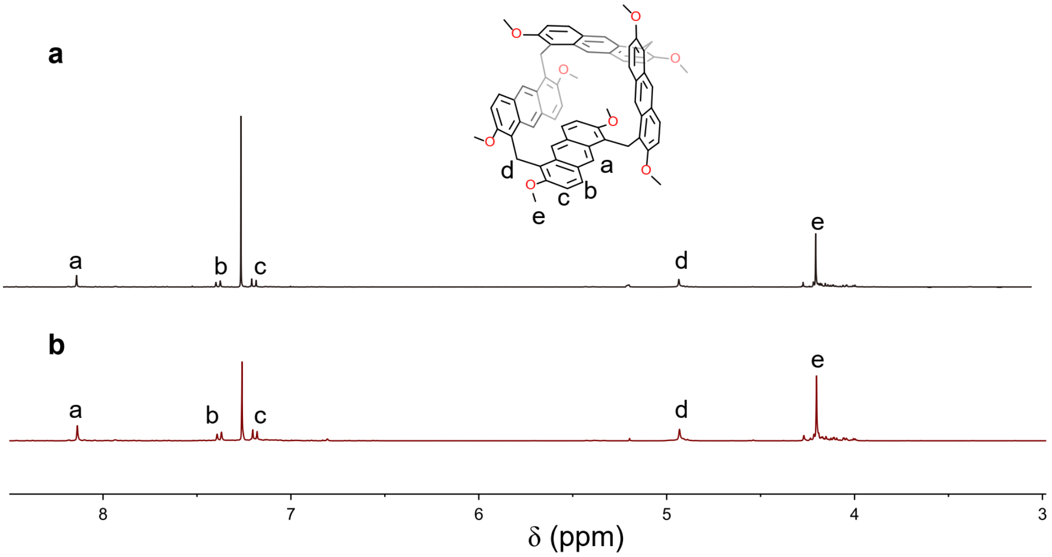

Supplementary Fig. 7. Stability tests for macrocycles under aqueous conditions. ^{1}H NMR spectra of P4 after immersion in water for two weeks (**a**) and **b**, P4 (400 MHz, $CDCl_3$).

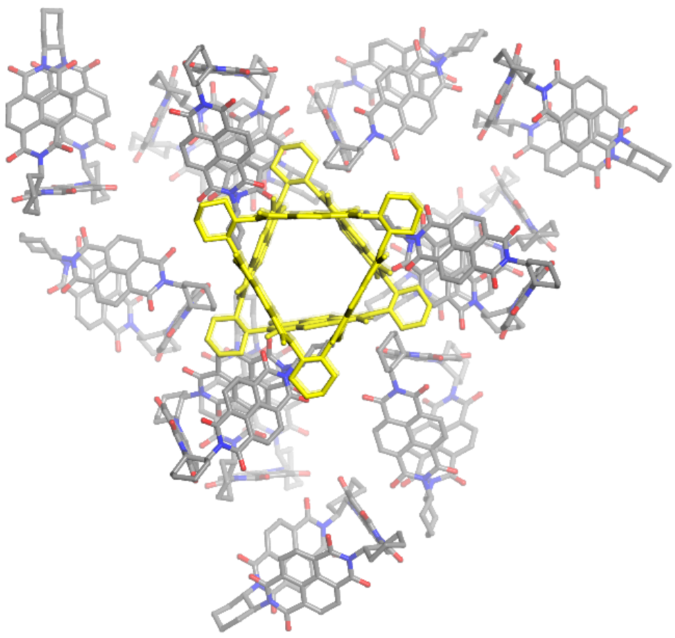

Supplementary Fig. 8. **Single crystal structure of (+)-NDI-△.** The tubular superstructure formed by (+)-NDI-△ is marked in yellow. Carbon atoms are shown in grey, nitrogen atoms are shown in blue, oxygen atoms are shown in red, and hydrogen atoms are omitted for clarity.

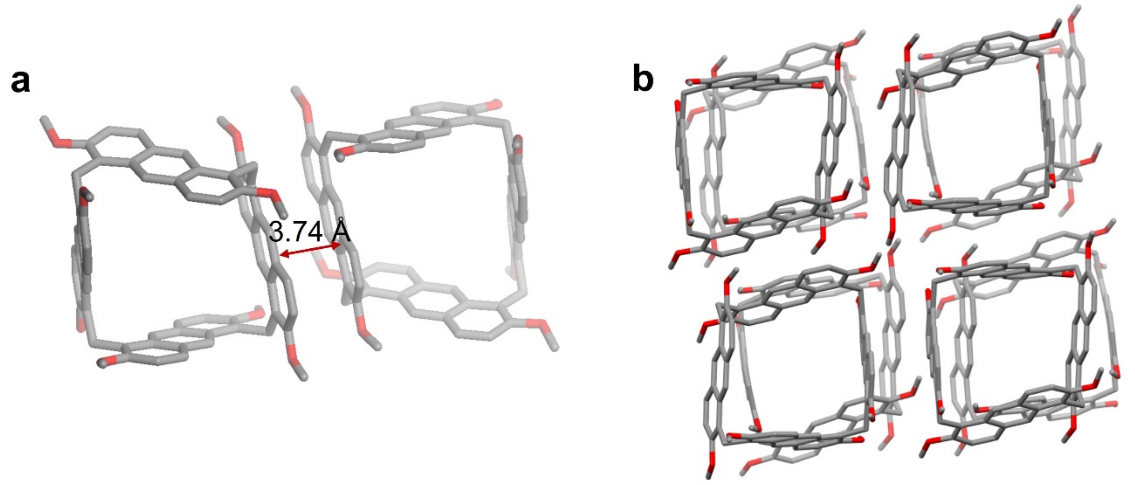

Supplementary Fig. 9. Single crystal structure of P4. a, π-π interaction between two neighbouring anthracene units of adjacent P4 molecules. **b**, Layer-by-layer packing structures of the 2D rhombic tessellations of P4. Carbon atoms are shown in grey, oxygen atoms are shown in red, and hydrogen atoms are omitted for clarity.

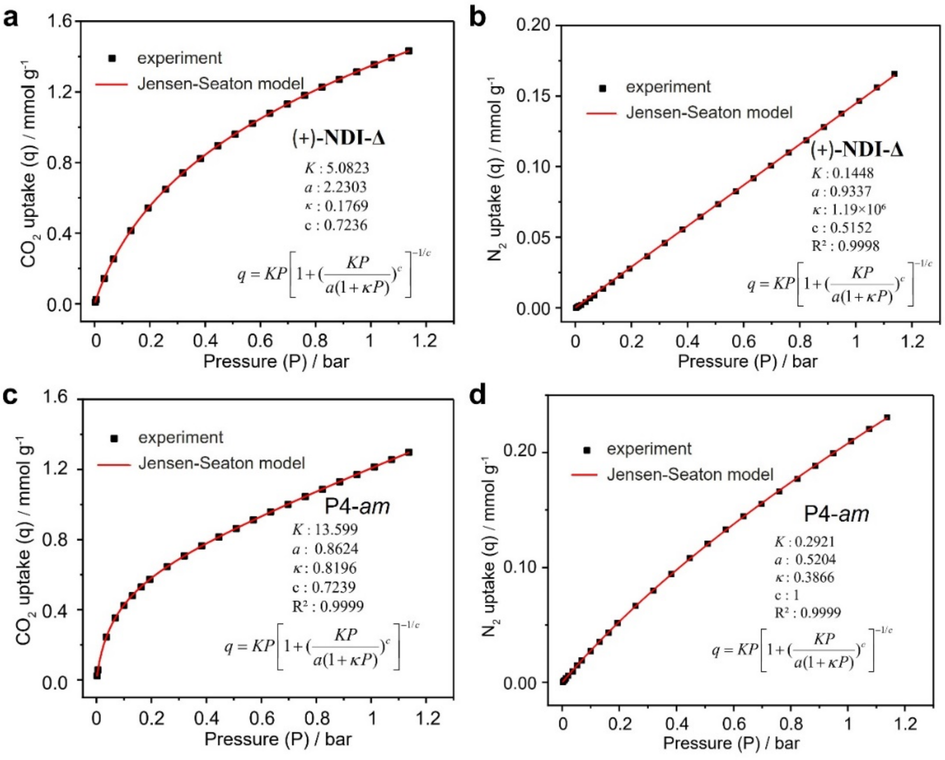

Supplementary Fig. 10. **Curve fitting for gas sorption isotherms.** Non-linear curve fits (Jensen-Seaton model) for CO_2 and N_2 sorption of (+)-NDI-Δ (**a**: CO_2; **b**: N_2) and P4-*am* (**c**: CO_2; **d**: N_2) at 298 K.

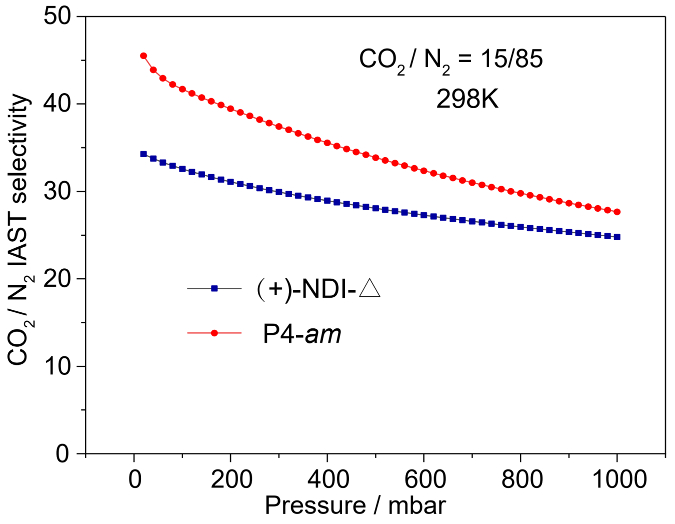

Supplementary Fig. 11. **IAST selectivity calculations.** Data were obtained for CO_2/N_2 = 15/85 mixtures for (+)-NDI-Δ (blue) and P4-*am* (red) at 298 K, as calculated from the pure CO_2 and N_2 gas sorption isotherms using pyGAPS.

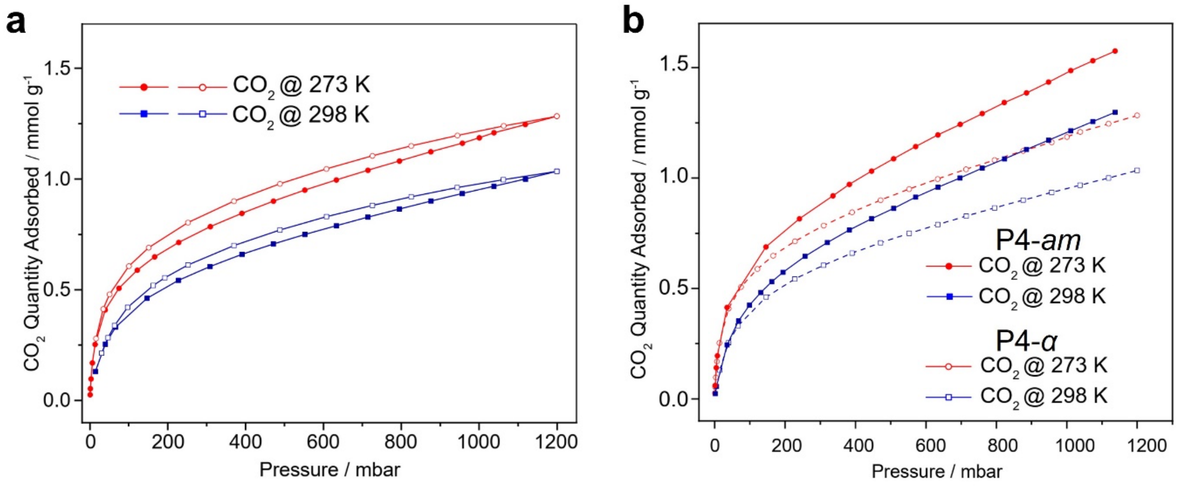

Supplementary Fig. 12. **CO_2 sorption isotherms for crystalline and amorphous P4. a**, CO_2 (273 K and 298K) sorption isotherms for crystalline P4. **b**, Comparison of CO_2 adsorption isotherms for crystalline (dash line) and amorphous (solid line) samples of P4.

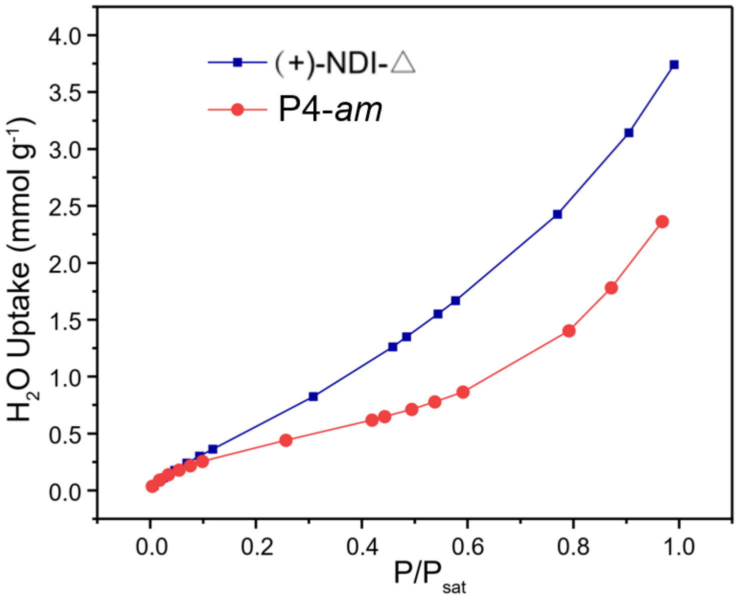

Supplementary Fig. 13. **Water sorption isotherms for the macrocycles.** Plot showing water adsorption for (+)-NDI-Δ (blue) and P4-*am* (red) at 298 K. These are among the lowest water uptakes reported thus far for any substantially porous solid.

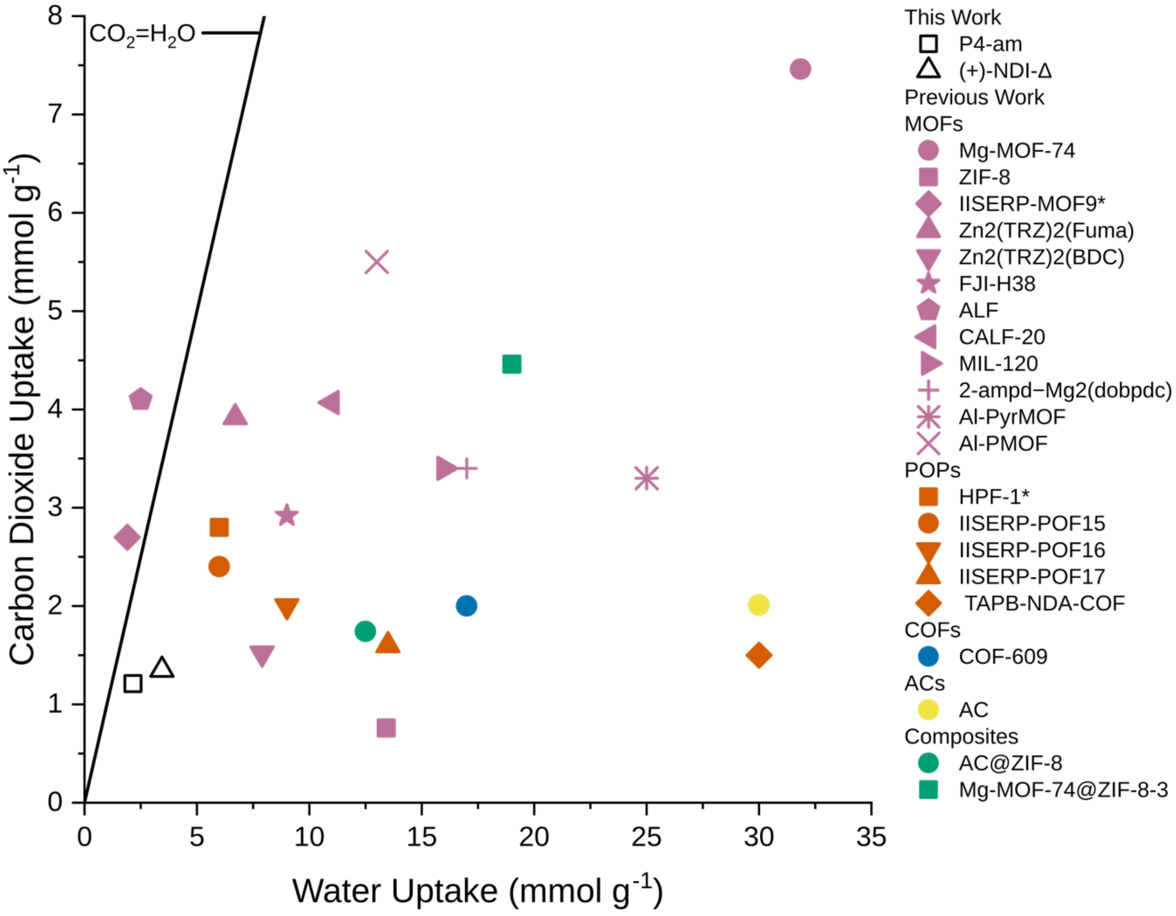

Supplementary Fig. 14. A comparison of materials with reported CO_2 (298 K, 1,000 mbar) and water (298 K, 32 mbar) isotherms in the literature. Plot of CO_2 uptake against water uptake of literature materials. Very few materials approach or cross the CO_2=H_2O line: almost all materials adsorb more water than CO_2 in a non-competitive comparison.

Table S1. Table of CO_2 uptake against water uptake of materials with reported CO_2 (298 K, 1,000 mbar) and water (298 K, 32 mbar) isotherms in the literature.

Material	Carbon Dioxide Uptake/ mmol g^{-1}	Water Uptake/ mmol g^{-1}	CO_2/H_2O	Reference
**P4-am**	1.21	2.16	0.56	This Work
**(+)-NDI-Δ**	1.35	3.45	0.39	This Work
**Mg-MOF-74**	7.46	31.85	0.23	[14]
**ZIF-8**	4.46	13.42	0.33	[14]
**IISERP-MOF9***	2.7	1.9	1.42	[15]
**Zn2(TRZ)2(Fuma)**	3.92	6.7	0.59	[16]
**Zn2(TRZ)2(BDC)**	1.52	7.9	0.19	[16]
**FJI-H38**	2.92	9	0.32	[17]
**ALF**	4.1	2.5	1.64	[18]
**CALF-20**	4.07	11	0.37	[19]
**MIL-120**	3.4	16	0.21	[20]
**2-ampd−Mg2(dobpdc)**	3.4	17	0.20	[21]

**Al-PyrMOF**	3.3	25	0.132	22
**Al-PMOF**	5.5	13	0.423	22
**HPF-1***	2.8	6	0.46	23
**IISERP-POF15**	2.4	6	0.40	24
**IISERP-POF16**	2	9	0.22	24
**IISERP-POF17**	1.6	13.5	0.11	24
**TAPB-NDA-COF**	1.5	30	0.05	25
**COF-609**	2	17	0.12	26
**AC**	2.01	30	0.07	27
**AC@ZIF-8**	1.74	12.5	0.14	27
**Mg-MOF-74@ZIF-8-3**	0.76	19.01	0.04	14

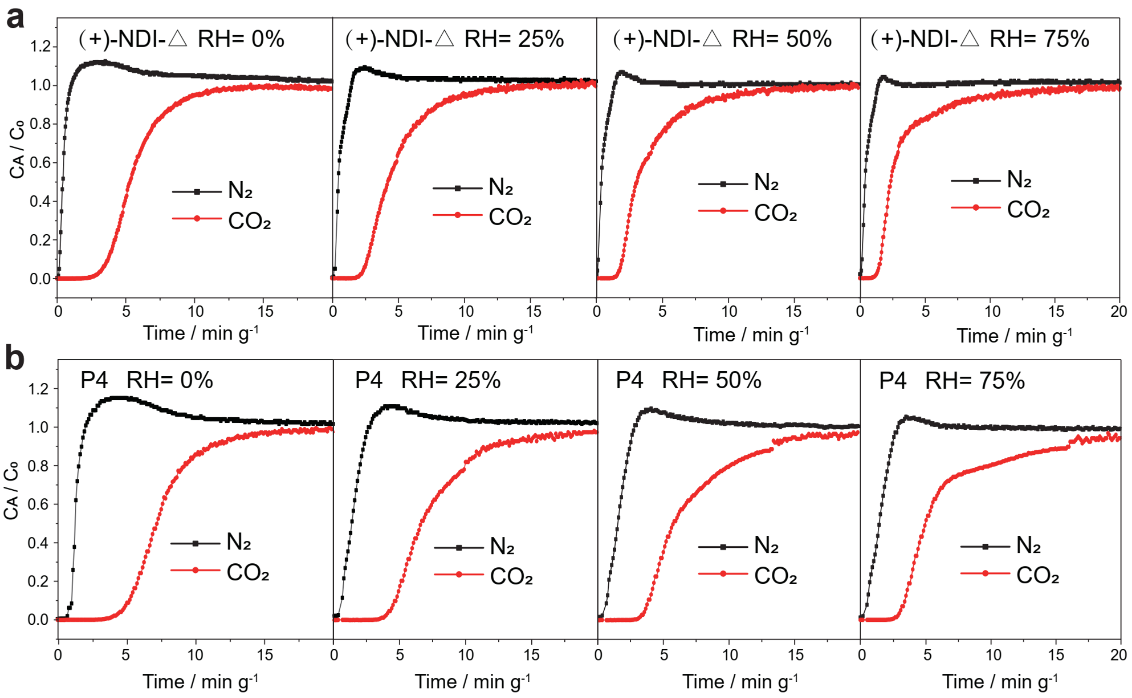

Supplementary Fig. 15. Experimental dynamic column breakthrough curves for macrocycles. Data are shown for **a**, (+)-NDI-Δ and **b**, P4-*am* (B) for CO_2/N_2 (15:85 v/v) separations at 298 K under different humidity conditions, ranging from 0% (dry) to 75% (from left to right).

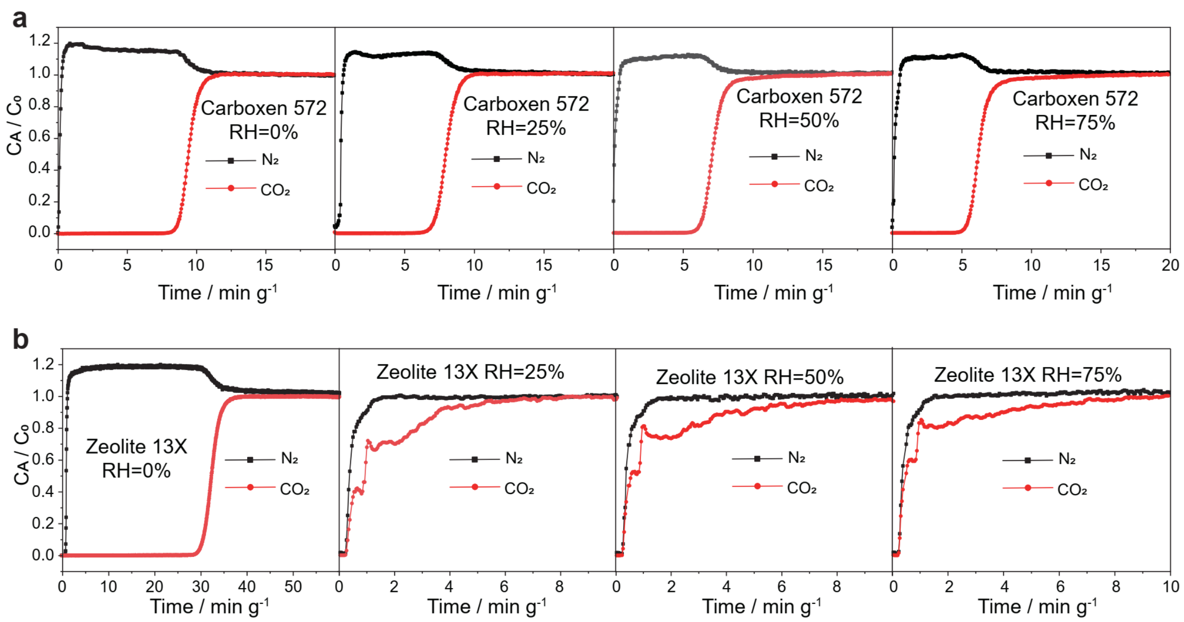

Supplementary Fig. 16. Experimental dynamic column breakthrough curves for benchmark materials. a, Carboxen 572 (a commercial activated carbon) and **b**, zeolite 13X (B) for CO_2/N_2 (15:85) separations at 298 K under different humidity conditions ranging from 0% (dry) to 75% (from left to right).

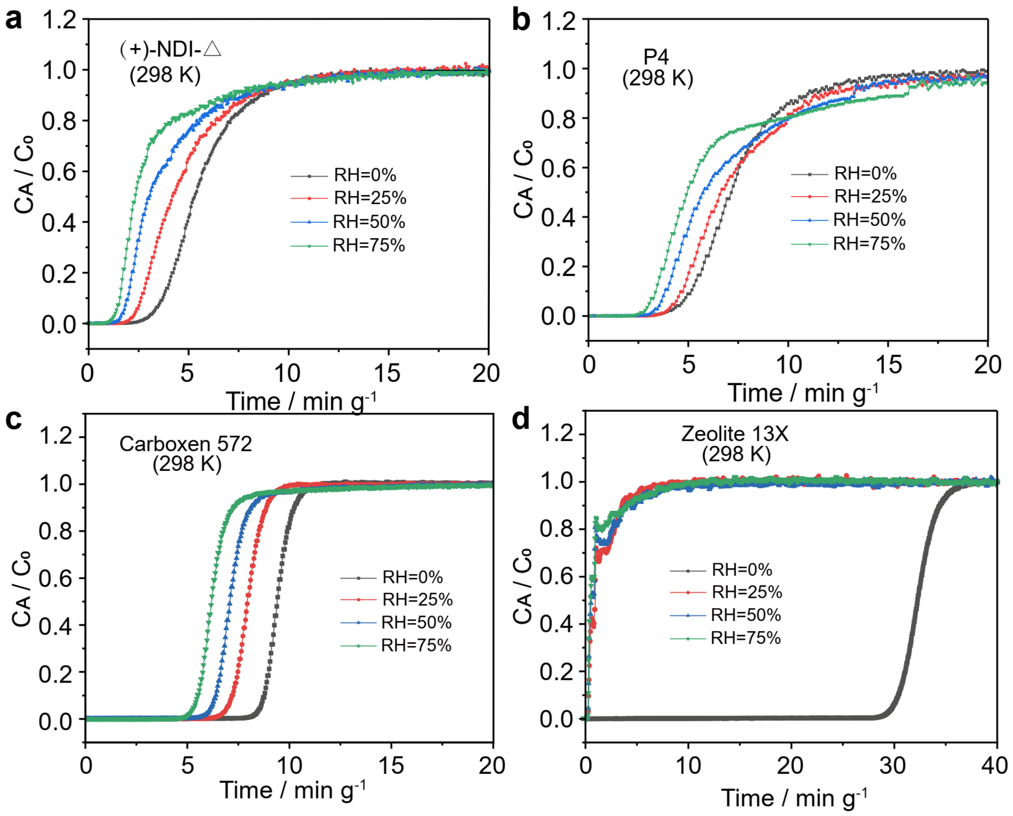

Supplementary Fig. 17. Comparison of CO_2 breakthrough curves for macrocycles and benchmark materials. a, (+)-NDI-Δ, **b**, P4-*am*, **c**, Carboxen 572 and **d**, zeolite 13X in dynamic column breakthrough experiments (CO_2 : N_2=15:85) at 298 K under different humidity conditions ranging from 0% to 75%.

Table S2. Summary of CO_2 capture capacity of (+)-NDI-Δ, P4-*am*, Carboxen 572 and zeolite 13X in dynamic column breakthrough experiments (CO_2 : N_2 = 15:85) at 298K under different humidity condition.

Samples	dry	RH=25%	RH=50%	RH=75%
(+)-NDI-Δ	0.37	0.31	0.27	0.24
P4-*am*	0.48	0.48	0.47	0.46
Carboxen 572	0.54	0.52	0.45	0.39
zeolite 13X	3.13	0.12	0.12	0.12

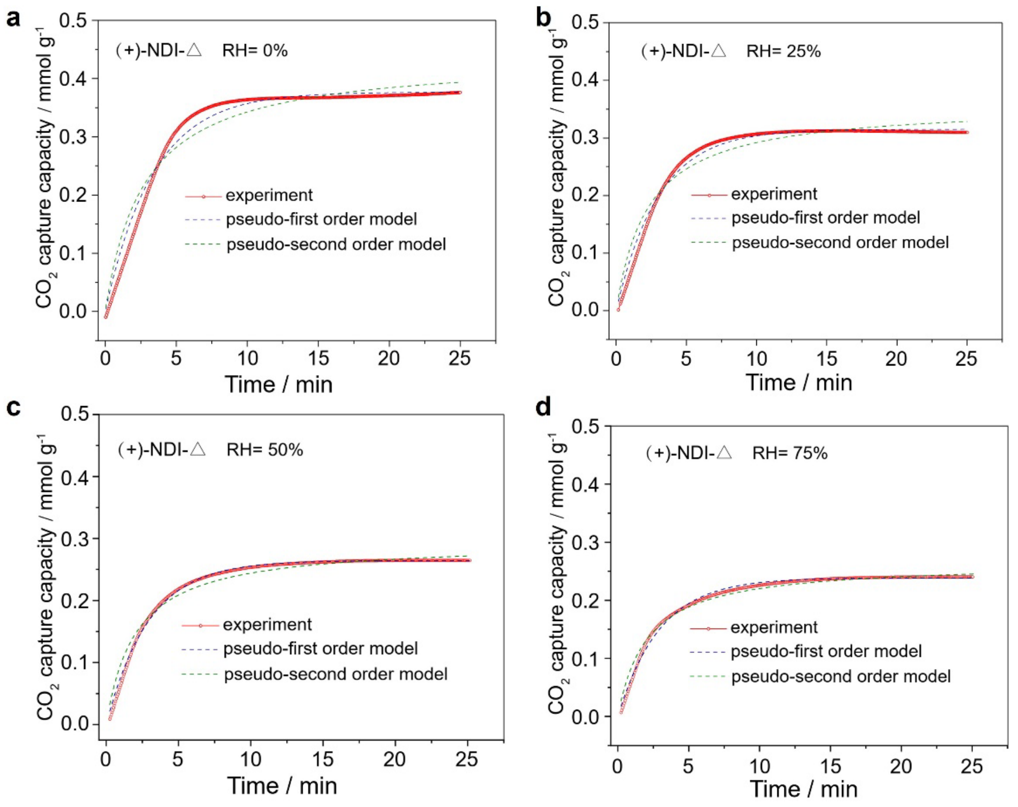

Supplementary Fig. 18. Kinetic fitting of adsorption profiles for (+)-NDI-Δ. Adsorption kinetic fitting for CO_2 working capacity of (+)-NDI-Δ in dynamic column breakthrough experiments (**a**, dry; **b**, RH=25%; **c**, RH=50%: **d**, RH=75% using pseudo-first order or pseudo-second order models.

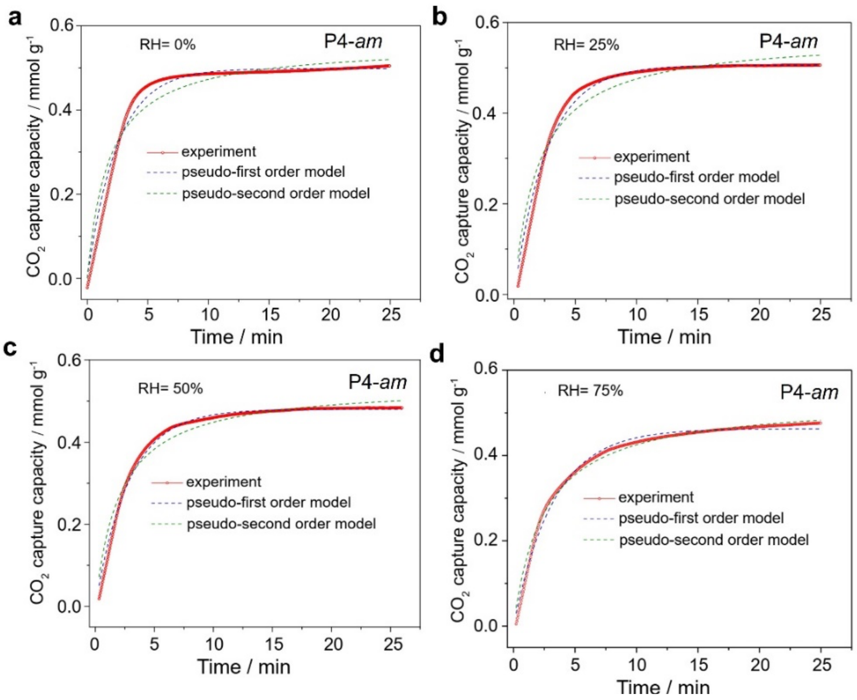

Supplementary Fig. 19. Kinetic fitting of adsorption profiles for P4-*am* Adsorption kinetic fitting for CO_2 working capacity of P4-*am* in dynamic column breakthrough experiments (**a**, dry; **b**, RH=25%; **c**, RH=50%; **d**, RH=75%) using pseudo-first order or pseudo-second order models.

Table S3. Adsorption kinetics fitting parameters of CO_2 capture capacity of (+)-NDI-Δ and P4-*am*.

Samples	Pseudo-first order model			Pseudo-second order model		
	q_e / mmol g^{-1}	k_1 / min^{-1}	R^2	q_e / mmol g^{-1}	k_2 / g·(mmol· min)$^{-1}$	R^2
(+)-NDI-Δ (dry)	0.377	0.2953	0.9773	0.437	0.8305	0.9334
(+)-NDI-Δ (25%)	0.315	0.3320	0.9861	0.359	1.2183	0.9345
(+)-NDI-Δ (50%)	0.264	0.3407	0.9962	0.293	1.6698	0.9617
(+)-NDI-Δ (75%)	0.239	0.3373	0.9930	0.265	1.8467	0.9776
P4-*am* (dry)	0.498	0.4061	0.9775	0.557	1.0076	0.9279
P4-*am* (25%)	0.506	0.3696	0.9845	0.570	0.8813	0.9346
P4-*am* (50%)	0.479	0.3567	0.9918	0.517	1.1142	0.9354
P4-*am* (75%)	0.462	0.3088	0.9901	0.531	0.7570	0.9870

Kinetic fitting of CO2 dynamic adsorption indicates that both (+)-NDI-△ and P4-am exhibit physical adsorption behaviours with pseudo-first-order kinetics (Supplementary Figs. 18 and 19 and Table 3). (+)-NDI-△ has a larger adsorption rate constant (~0.34 min-1) under humid condition compared to dry conditions (~0.30 min-1). By contrast, the CO2 adsorption rate constant of P4-am decreases from 0.40 to 0.30 min-1 with increasing relative humidity (Supplementary Table 3), indicating that the diffusion resistance of CO_2 increases due to the displacement of water within the cavities of P4.

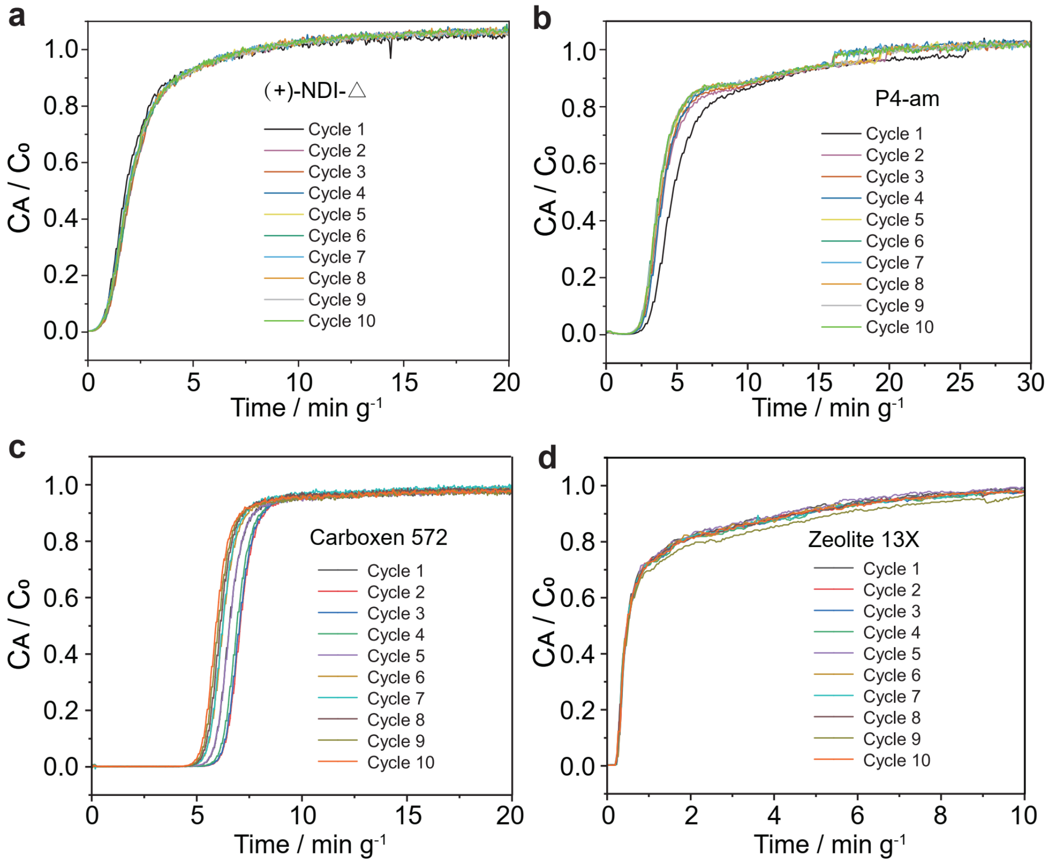

Supplementary Fig. 20. Repeated cycling experiments (10 cycles) of (+)-NDI-Δ (**a**), P4-*am* (**b**), Carboxen 572 (**c**) and zeolite 13X (**d**) under humid dynamic column breakthrough tests (RH=75%, CO_2:N_2=15:85, 298K).

Table S4. CO_2 capture capacity of (+)-NDI-Δ, P4-*am*, Carboxen 572 and zeolite 13X in repeated cycling experiments (10 cycles).

Samples	dry	1	2	3	4	5	6	7	8	9	10
(+)-NDI-Δ	0.37	0.24	0.24	0.24	0.24	0.24	0.24	0.24	0.24	0.24	0.24
P4	0.48	0.46	0.43	0.43	0.43	0.43	0.43	0.43	0.43	0.43	0.43
Carboxen 572	0.54	0.36	0.35	0.35	0.34	0.33	0.33	0.31	0.31	0.31	0.30
zeolite 13X	3.13	0.12	0.12	0.12	0.12	0.12	0.12	0.12	0.12	0.12	0.12

3. Synthetic procedures and NMR data for (+)-NDI-Δ and P4

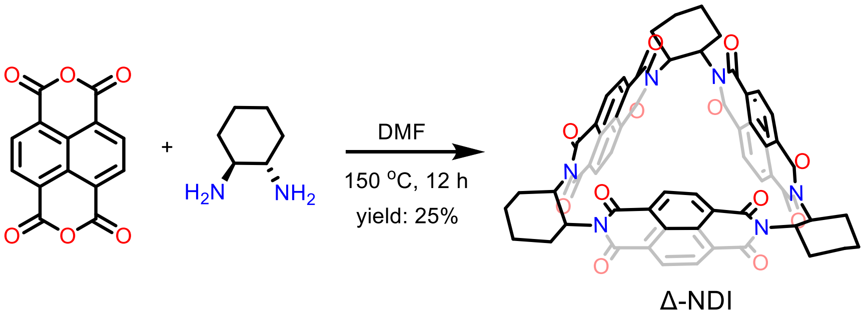

Synthesis of (+)-NDI-Δ: A solution of (S, S)-trans-1, 2-cyclohexanediamine [(S, S)-CHDA, 0.88 g, 7.5 mmol] in DMF (10 ml) was added rapidly to a solution of naphthalenetetracarboxylic dianhydride (1.49 g, 7.4 mmol) in DMF (100 ml) with vigorous stirring. The mixture was refluxed at 160 °C for 12 h. After reaction completed, DMF was evaporated to dryness under reduced pressure (~3 mbar) at 75 °C. The crude product was purified by column chromatography over silica gel using CH_2Cl_2/acetone (0-10% vol.). The crude product was recrystalized by using CH_2Cl_2 and MeOH to afford pure (+)-NDI-Δ (0.60 g) in 24% yield as a dark red solid. ^{1}H NMR (400 MHz, $CDCl_3$): δ 8.48 (d, J = 1.6 Hz, 12H), 6.23 (d, J = 9.6 Hz, 6H), 2.49 (d, J = 11.0 Hz, 4H), 2.03-1.91 (m, 14H), 1.67 (t, J = 10.1 Hz, 6H). ^{13}C NMR (100 MHz, $CDCl_3$) δ 162.75, 162.46, 131.35, 130.75, 126.48, 126.11, 125.85, 53.92, 29.96, 25.75.

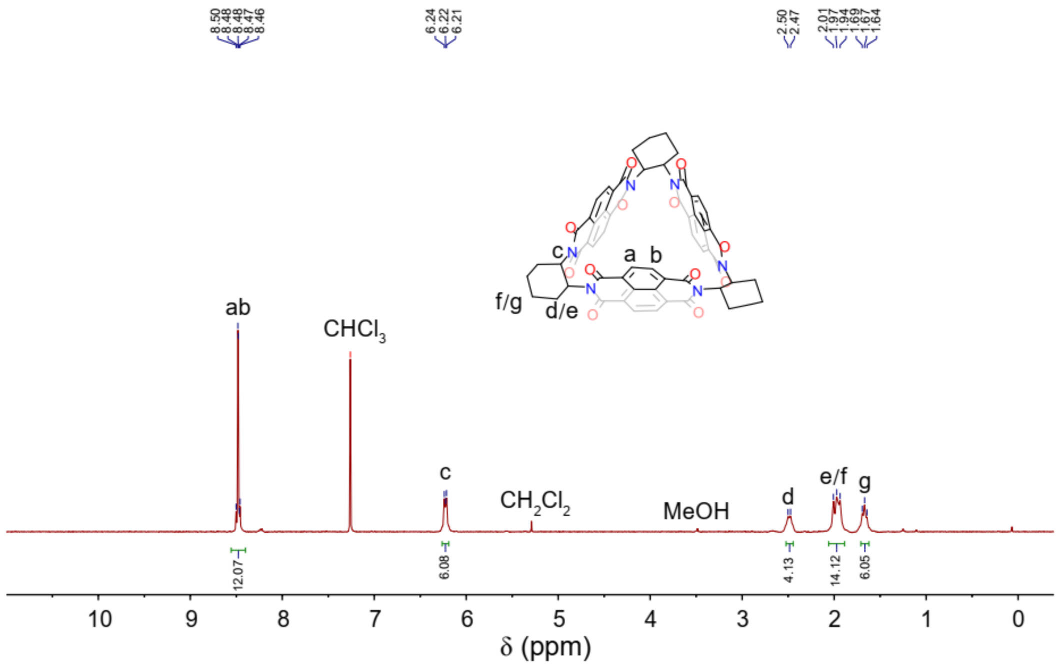

Supplementary Fig. 21. ^{1}H NMR spectrum of (+)-NDI-Δ (400 MHz, $CDCl_3$).

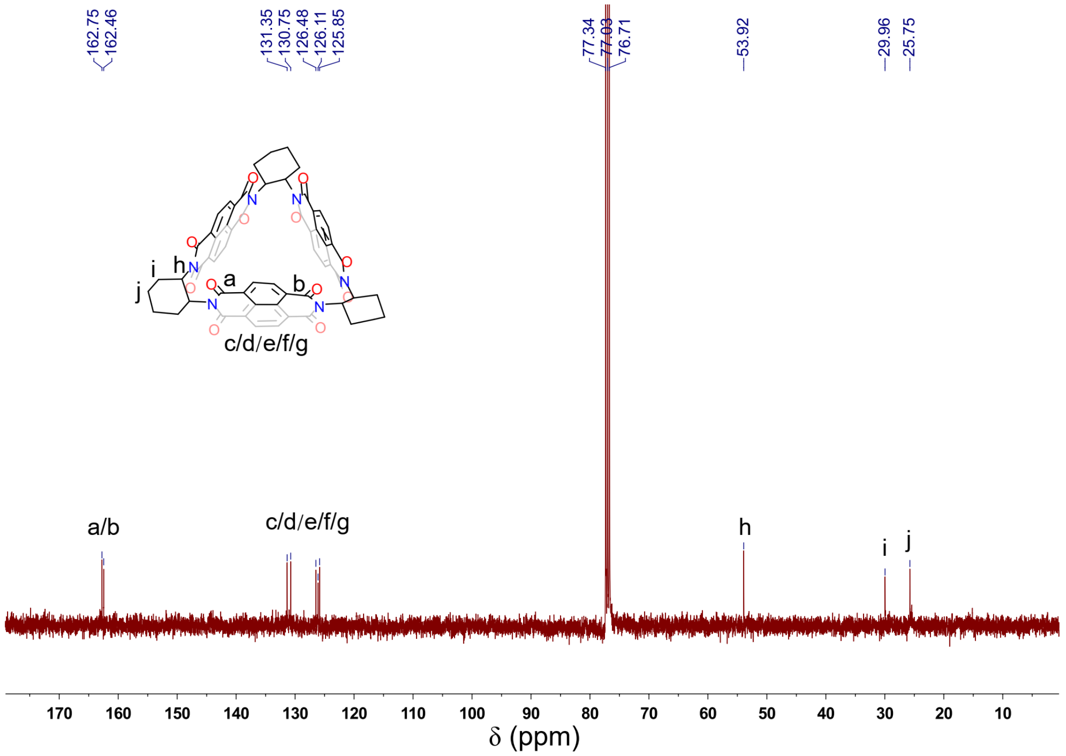

Supplementary Fig. 22. ^{13}C NMR spectrum of (+)-NDI-Δ (100 MHz, $CDCl_3$).

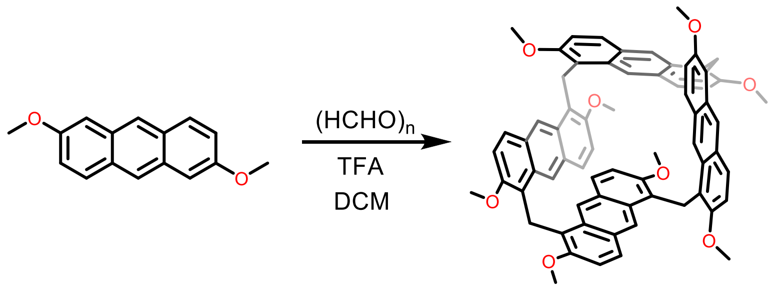

Synthesis of P4: The mixture of 2,6-dimethoxyanthracene (1.8 g, 7.5 mmol) and paraformaldehyde (705 mg, 22.5 mmol) was dissolved in dichloromethane (600 mL). The solution was bubbled using N_2 for 0.5 h and then was added TFA (304 μL, 4 mmol). After the mixture was stirred at room temperature under N_2 atmosphere for 12 h, water (400 mL) was added to the flask to quench the reaction. The organic phase was separated and dried over anhydrous sodium sulphate and then evaporated to dryness. The residue was purified by column chromatography on silica gel (eluent: 1:1 petroleum ether/EA) to give P4 (521 mg, 27%) as yellow solids. ^{1}H NMR (400 MHz, CDCl3): δ 8.14 (s, 8H), 7.38 (d, J = 9.2 Hz, 8H), 7.19 (d, J = 9.3 Hz, 8H), 4.93 (s, 8H), 4.20 (s, 24H). ^{13}C NMR (100 MHz, CDCl3): δ 152.09, 129.89,129.16,128.59, 123.32, 122.06, 114.24, 57.19, 20.80.

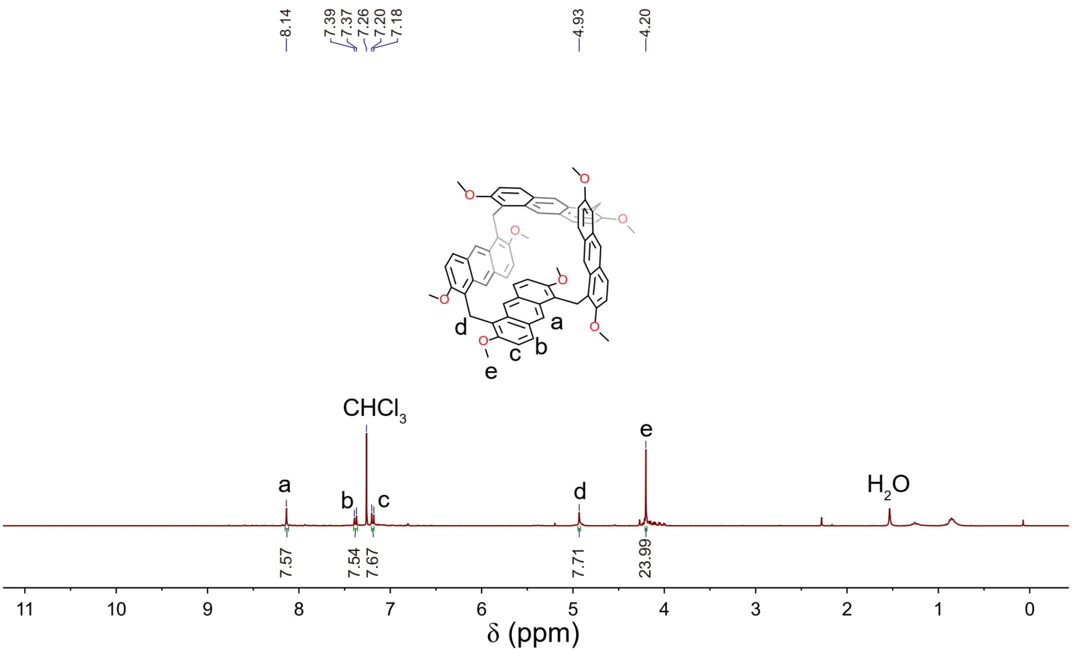

Supplementary Fig. 23. ^{1}H NMR spectrum of P4 (400 MHz, $CDCl_3$).

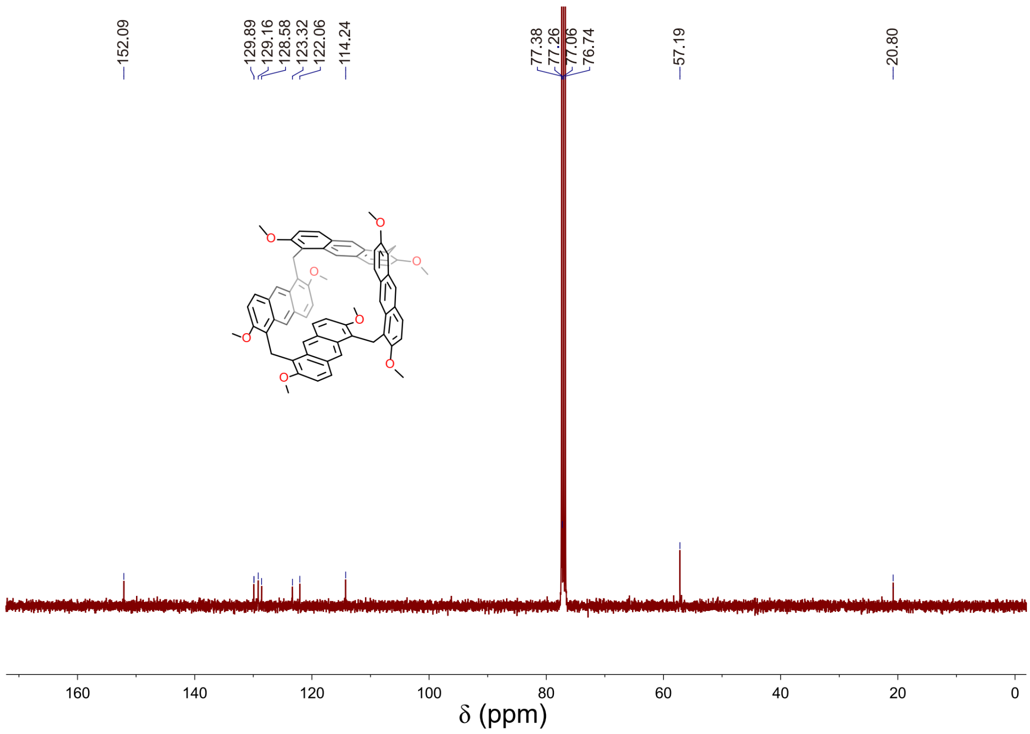

Supplementary Fig. 24. ^{13}C NMR spectrum of P4 (100 MHz, $CDCl_3$).

Table S5. Crystal data and structure refinements

	(+)-NDI-Δ	P4
Empirical formula	$C_{60}H_{42}N_6O_{12}$	$C_{68}H_{56}O_8 + 2CH_2Cl_2$
Formula weight	1038.99	1170.98
Temperature (K)	100	171
Crystal system	cubic	monoclinic
Space group	$I2_13$	$I2/a$
a(Å)	29.3836(5)	15.9992(3)
b(Å)	29.3836(5)	16.8582(4)
c(Å)	29.3836(5)	21.2671(3)
α (deg)	90	90
β (deg)	90	105.685(2)
γ (deg)	90	90
V(Å^3)	25369.8(13)	5522.51(19)
Z	16	4
ρ_{cal} (g·m^{-3})	1.088	1.408
μ(mm^{-1})	0.077	2.443
F(000)	8640.0	2448.0
Crystal size (mm)	0.2×0.2×0.1	0.2×0.2×0.1
Radiation	Mo Kα (λ = 0.71073 Å)	Cu Kα (λ =1.54184 Å)
2Θ range for data collection (deg)	3.396 to 52.722	6.792 to 160.508
Index ranges	$-36 \le h \le 36$, $-36 \le k \le 30$, $-24 \le l \le 3$	$-20 \le h \le 20$, $-21 \le k \le 20$, $-26 \le l \le 17$
Reflections collected	85929	27042
Independent reflections	8644 [R_{int} = 0.0587, R_{sigma} = 0.0318]	5873 [R_{int} = 0.0462, R_{sigma} = 0.0293]
Data/restraints/parameters	8644/23/471	5873/12/375
Goodness-of-fit on F^2	0.979	1.057
Final R indexes [$I>2\sigma(I)$]	R_1 = 0.0684, wR_2 = 0.2073	R_1 = 0.0779, wR_2 = 0.2148
Final R indexes (all data)	R_1= 0.1104, wR_2 = 0.2502	R_1 = 0.0854, wR_2 = 0.2222
Largest diff. peak/hole(e Å^{-3})	0.21/-0.23	0.58/-0.51
CCDC#	2384534	2384535

Crystallographic data have been submitted to the Cambridge Crystallographic Database with according reference numbers and are available free of charge at http://www.ccdc.cam.ac.uk/data_request/cif

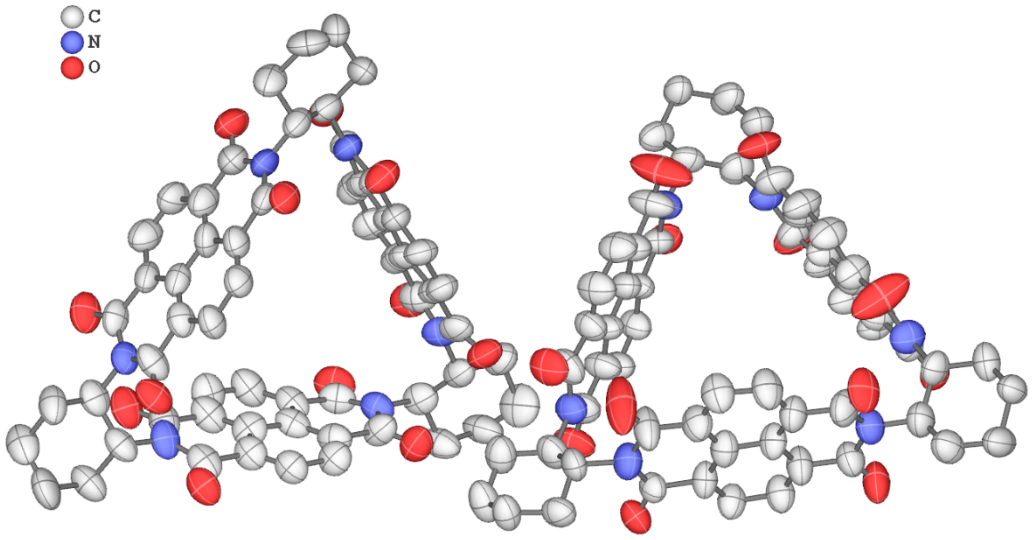

Supplementary Fig. 25. Displacement ellipsoid plot for all non-H atoms in the single crystal structure of **(+)-NDI-Δ** (at 50% probability level).

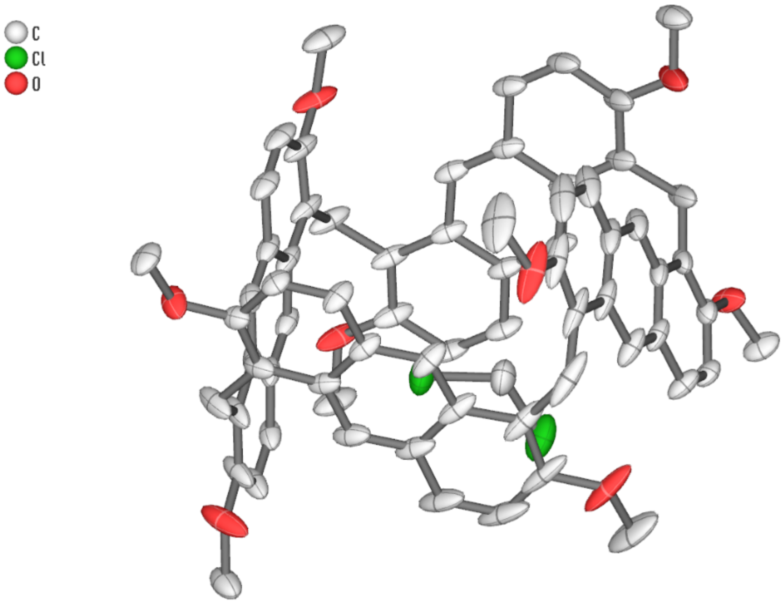

Supplementary Fig. 26. Displacement ellipsoid plot for all non-H atoms in the single crystal structure of **P4** (at 50% probability level).

Supplementary References

1. Grimme, S., Bannwarth, C. & Shushkov, P. A robust and accurate tight-binding quantum chemical method for structures, vibrational frequencies, and noncovalent interactions of large molecular systems parametrized for all spd-block elements (Z = 1–86). *J. Chem. Theory Comput.* **13**, 1989–2009 (2017).
2. Parr, R. G. & Weitao, Y. *Density-Functional Theory of Atoms and Molecules*. (Oxford University Press, 1995). doi:10.1093/oso/9780195092769.001.0001.
3. Grimme, S., Ehrlich, S. & Goerigk, L. Effect of the damping function in dispersion corrected density functional theory. *J. Comput. Chem.* **32**, 1456–1465 (2011).
4. Weigend, F. & Ahlrichs, R. Balanced basis sets of split valence, triple zeta valence and quadruple zeta valence quality for H to Rn: Design and assessment of accuracy. *Phys Chem Chem Phys* **7**, 3297–3305 (2005).
5. Frisch, M. J. *et al.* Gaussian 16 Rev. A.03. (2016).
6. Sheldrick, G. M. Crystal structure refinement with ıt SHELXL. *Acta Crystallogr. Sect. C* **71**, 3–8 (2015).
7. Dolomanov, O. V., Bourhis, L. J., Gildea, R. J., Howard, J. A. K. & Puschmann, H. OLEX2: a complete structure solution, refinement and analysis program. *J. Appl. Crystallogr.* **42**, 339–341 (2009).
8. Wilkins, N. S., Rajendran, A. & Farooq, S. Dynamic column breakthrough experiments for measurement of adsorption equilibrium and kinetics. *Adsorption* **27**, 397–422 (2021).
9. Weininger, D. SMILES, a chemical language and information system. 1. Introduction to methodology and encoding rules. *J. Chem. Inf. Comput. Sci.* **28**, 31–36 (1988).
10. Weininger, D., Weininger, A. & Weininger, J. L. SMILES. 2. Algorithm for generation of unique SMILES notation. *J. Chem. Inf. Comput. Sci.* **29**, 97–101 (1989).
11. Weininger, D. SMILES. 3. DEPICT. Graphical depiction of chemical structures. *J. Chem. Inf. Comput. Sci.* **30**, 237–243 (1990).
12. Rappe, A. K., Casewit, C. J., Colwell, K. S., Goddard, W. A. & Skiff, W. M. UFF, a full periodic table force field for molecular mechanics and molecular dynamics simulations. *J. Am. Chem. Soc.* **114**, 10024–10035 (1992).

13. Landrum, G. *et al.* rdkit/rdkit: 2022_03_5 (Q1 2022) Release. Zenodo https://doi.org/10.5281/zenodo.6961488 (2022).
14. Wan, Y. *et al.* Enhancing hydrophobicity via core–shell metal organic frameworks for high-humidity flue gas CO2 capture. *Chin. J. Chem. Eng.* **61**, 82–89 (2023).
15. Nandi, S., Haldar, S., Chakraborty, D. & Vaidhyanathan, R. Strategically designed azolyl-carboxylate MOFs for potential humid CO2 capture. *J Mater Chem A* **5**, 535–543 (2017).
16. Gu, Y.-M. *et al.* N-donating and water-resistant Zn-carboxylate frameworks for humid carbon dioxide capture from flue gas. *Fuel* **336**, 126793 (2023).
17. Song, D., Jiang, F., Yuan, D., Chen, Q. & Hong, M. Optimizing Sieving Effect for CO2 Capture from Humid Air Using an Adaptive Ultramicroporous Framework. *Small* **19**, 2302677 (2023).
18. Evans, H. A. *et al.* Aluminum formate, Al(HCOO)3: An earth-abundant, scalable, and highly selective material for CO2 capture. *Sci. Adv.* **8**, eade1473.
19. Lin, J.-B. *et al.* A scalable metal-organic framework as a durable physisorbent for carbon dioxide capture. *Science* **374**, 1464–1469 (2021).
20. Loughran, R. P. *et al.* CO2 capture from wet flue gas using a water-stable and cost-effective metal-organic framework. *Cell Rep. Phys. Sci.* **4**, 101470 (2023).
21. Siegelman, R. L. *et al.* Water Enables Efficient CO2 Capture from Natural Gas Flue Emissions in an Oxidation-Resistant Diamine-Appended Metal–Organic Framework. *J. Am. Chem. Soc.* **141**, 13171–13186 (2019).
22. Boyd, P. G. *et al.* Data-driven design of metal–organic frameworks for wet flue gas CO2 capture. *Nature* **576**, 253–256 (2019).
23. Nandi, S., Werner-Zwanziger, U. & Vaidhyanathan, R. A triazine–resorcinol based porous polymer with polar pores and exceptional surface hydrophobicity showing CO2 uptake under humid conditions. *J. Mater. Chem.* **3**, 21116–21122 (2015).
24. Singh, H. D., Singh, P., Rase, D. & Vaidhyanathan, R. Pore volume regulated CO2 adsorption in C–C bonded porous organic frameworks. *Mater Adv* **4**, 3055–3060 (2023).

25. Veldhuizen, H. *et al.* Competitive and Cooperative CO2–H2O Adsorption through Humidity Control in a Polyimide Covalent Organic Framework. *ACS Appl. Mater. Interfaces* **15**, 29186–29194 (2023).

26. Lyu, H., Li, H., Hanikel, N., Wang, K. & Yaghi, O. M. Covalent Organic Frameworks for Carbon Dioxide Capture from Air. *J. Am. Chem. Soc.* **144**, 12989–12995 (2022).

27. Ji, Y. *et al.* Hydrophobic ZIF-8 covered active carbon for CO2 capture from humid gas. *J. Ind. Eng. Chem.* **121**, 331–337 (2023).
